# Cancer treatment approaches within the frame of hyperthermia, drug delivery systems, and biosensors: concepts and future potentials

**DOI:** 10.1039/d4ra06992g

**Published:** 2024-12-12

**Authors:** Zeinab S. Sayed, Eman M. Hieba, Hany A. Batakoushy, Huda R. M. Rashdan, Enas Ismail, Saeid M. Elkatlawy, Amir Elzwawy

**Affiliations:** a Faculty of Applied Medical Science, Misr University for Science and Technology (MUST) Giza Egypt; b Chemistry and Entomology Department, Faculty of Science, Cairo University Giza 12613 Egypt; c Department of Pharmaceutical Analytical Chemistry, Faculty of Pharmacy, Menoufia University Shebin Elkom 32511 Egypt; d Chemistry of Natural and Microbial Products Department, Pharmaceutical and Drug Industries Research Institute, National Research Centre 33 El Buhouth St., Dokki Giza 12622 Egypt Hudadawoud20@yahoo.com; e Department of Prosthodontics, Faculty of Dentistry, University of the Western Cape Cape Town 7505 South Africa; f Physics Department, Faculty of Science (Girl's Branch), Al Azhar University Nasr City 11884 Cairo Egypt; g Department of Physics, Faculty of Science, University of Sadat City Fifth Zone Sadat Egypt; h Ceramics Department, Advanced Materials Technology and Mineral Resources Research Institute, National Research Centre (NRC) 33 El Bohouth St., Dokki Giza 12622 Egypt elzwawy1@gmail.com aa.elzwawy@nrc.sci.eg

## Abstract

This work presents a review of the therapeutic modalities and approaches for cancer treatment. A brief overview of the traditional treatment routes is presented in the introduction together with their reported side effects. A combination of the traditional approaches was reported to demonstrate an effective therapy until a few decades ago. With the improvement in the fabrication of nanomaterials, targeted therapy represents a novel therapeutic approach. This improvement established on nanoparticles is categorized into hyperthermia, drug delivery systems, and biosensors. Hyperthermia presents a personalized medicine-based approach in which targeted zones are heated up until the diseased tissue is destroyed by the thermal effect. The use of magnetic nanoparticles further improved the effectiveness of hyperthermia owing to the enhanced heating action, further increasing the accuracy of the targeting process. Nanoparticle-based biosensors present a smart nanodevice that can detect, monitor, and target tumor tissues by following the biomarkers in the body fluids. Magnetic nanoparticles offer a controlled thermo-responsive device that can be manipulated by changing the magnetic field, offering a more personalized and controlled hyperthermia therapeutic modality. Similarly, gold nanoparticles offer an effective aid in the hyperthermia treatment approach. Furthermore, carbon nanotubes and metal–organic frameworks present a cutting-edge approach to cancer treatment. A combination of functionalized nanoparticles offers a unique route for drug delivery systems, in which therapeutic agents carried by nanoparticles are guided into the human body and then released in the target spot.

## Abstract 2

This work presents a review of the therapeutic modalities and approaches for cancer treatment.

## Graphical abstract

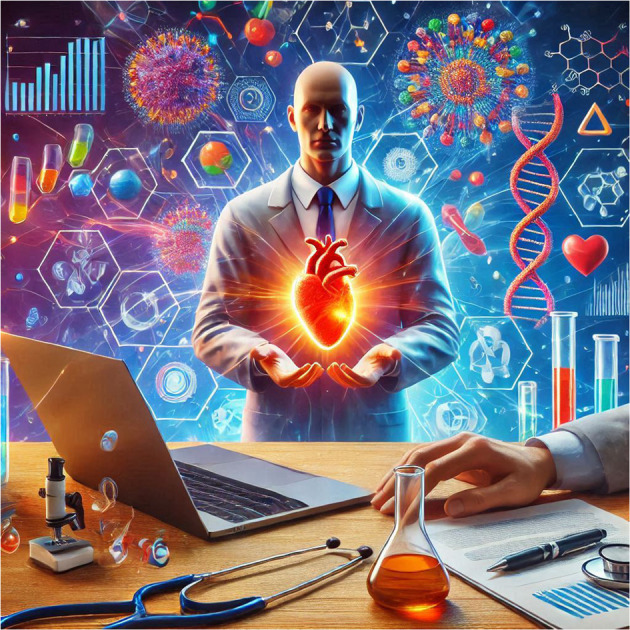

## 1. Introduction

Scientific research always opens the doors to novel routes for improving the quality of life of human beings. Cancer is a global health issue and leading cause of poor life quality and death worldwide. It is characterized by abnormal spread and uncontrolled growth of biological cells.^1,2^ Cancer is a complex and diverse disease characterized by many genetic and molecular changes that cause the unregulated growth and multiplication of cells, resulting in the rapid accumulation of tissues in the affected areas of the body. Typically, a cell receives instructions to undergo programmed cell death and be replaced with a younger and healthier cell.^3^

The history of cancer detection and treatment dates back to ancient Egyptians and Greeks,^4^ where they treated tumors by surgery. However, this was not an effective approach and led to the death of the diseased patient. Later, the discovery of X-rays and the manufacturing of anti-tumor drugs paved the way for novel approaches in the detection and treatment of cancer.^5^ One of the most popular cancer treatment approaches is chemotherapy, in which specialized drugs are used to either kill cancer cells or prevent them from proliferating. In this treatment modality, the drug can be injected intramuscularly or administered intravenously or orally. Physicians sometimes use surgery or radiation therapy in combination with chemotherapy as a complementary treatment approach.

In radiation therapy, oriented doses of high-energy particles, such as gamma rays, X-rays, protons, or electron beams, are applied to kill tumor cells. This treatment modality is sometimes associated with harm to surrounding healthy tissues; to minimize this harm, a concentrated beam is targeted to the precise spot of tumor tissue. However, both chemotherapy and radiotherapy have serious side effects, such as hair loss, continuous fatigue, and nausea.^6–8^ With attention to better treatment quality and less harm to patients, scientists are always looking for different treatment routes. Targeted drug delivery systems, biosensors, and hyperthermia are considered effective approaches and less harmful compared to traditional surgery, chemotherapy and radiotherapy. They offer quality treatment modalities and are best known for their adequacy and reduced side effects.^1^ In the era of nanotechnology and novel architected nanoparticles, examples of targeted therapies are hyperthermia which involves the use of heat to kill cancer cells,^9,10^ controlled drug delivery systems that aim to deliver drugs directly to the tumor site to minimize side effects,^11–13^ and biosensors for the detection and monitoring of cancer cells for effective targeted treatment.^14–17^ These treatment routes aim to target the diseased tumor cells precisely to reduce the side effects on normal cells resulting in an effective therapy. These various therapeutic modalities have increased the chances of cancer survival considerably and provided new opportunities for cancer research.

In hyperthermia, biosensors, or combined modality of both together with drug delivery systems, functionalized nanoparticles with a thermo-responsive effect are employed accurately to target the diseased tumor tissue. These nanoparticles, which can be fabricated using special techniques, show promise as on-demand drug delivery systems due to their controlled physicochemical properties that meet the requirements of hyperthermia treatments. Furthermore, the use of magnetic nanomaterials in magnetic hyperthermia therapy offers an economic and sustainable way to use an alternating magnetic field to heat nanoparticles in tumor tissues.^18–20^

The investigation of the cytotoxicity of nanomaterials is essential for medical applications including cancer therapy. This involves *in vitro* cell culture tests to evaluate the toxicity thresholds, biodistribution, and cell proliferation rates. It is vital to study the molecular mechanisms of their toxicity. Different factors can affect the nanomaterial toxicity including surface modifications, coating, and the formation of protein around NPs in biological fluids. Hence, developing nanomaterials with minimal cytotoxic effects and high therapeutic benefits is a complex challenge that requires interdisciplinary approaches.^21–23^

Nano-biosensors are used in combination with targeted therapy, and they provide a promising approach for accurate detection and monitoring of the treatment progression. The use of magnetic nanoparticles as biosensors allows for precise concentrations to act on diseased tissues while enabling to accurately increase the tissue temperature. This modality combined with the hyperthermia approach yields effective treatment.^24–26^ Nano-biosensors are designed with the ability to identify specific biomarkers linked to different types of tumor tissues, providing a potent diagnosis and detection tool. These nanodevices can quickly quantify the chemicals linked to tumor tissues in different body fluids, giving rise to specific diagnoses and customized treatment protocols. Due to the real-time monitoring and control of biosensors, this approach can minimize damage to healthy tissues while delivering therapeutic agents to diseased tissues.^27–29^

In this review, the authors report on novel approaches to cancer treatment for improving the quality of life and decreasing risk factors that surround cancer patients.

### 1.1 Side effects of cancer therapy

Due to demographic shifts, environmental degradation, and the increasing frequency of risk factors including unhealthy lifestyle, it is anticipated that the global incidence of cancer will increase significantly in the next two decades.^30^ The concept of chemotherapy, which involves the use of hazardous substances and medications to eliminate cancer cells, originated from the observation of mustard gas destroying lymphatic tissues and bone marrow. Aggressive tumors are characterized by uncontrolled and widespread cell division, which can disrupt the normal functioning of nearby non-transformed cells in the affected organs or tissues. Cancer cells possess highly developed metabolism and survival abilities that enable them to withstand harsh conditions such as limited oxygen and nutrition availability.^31^ Therefore, there is an urgent need for effective medical interventions to decrease the total death rate caused by cancer. Traditional cancer treatments, including surgical procedures, chemotherapy, and radiation therapy, have effectively enhanced the survival rates of many patients. Nevertheless, their effectiveness in treating advanced metastatic malignancies is limited.^32^ The diverse composition of tumor mass significantly contributes to the development of medication resistance which presents a unique issue, since doctors are left with little choice but to alter the prescription periodically when a particular drug becomes ineffective against tumors. Combination treatment, which involves the simultaneous use of many drugs, can be a highly effective approach in treating certain types of cancers such as leukemia and lymphoma, which affect the bone marrow and lymph nodes, respectively. The sustainability of chemotherapy depends on its ongoing impact on cell division and the growth of tumors. Most therapies function by destabilizing or degrading the stability and integrity of nucleic acids, which can lead to cell cycle arrest or activation of programmed cell death pathways. Some medications can cause severe proteotoxic stress within cells by disrupting the protein folding or degradation processes, which involve chaperones, proteasomes, and autophagy.^33^

Immunotherapies, while being a significant advancement in the treatment of advanced malignancies, are hindered by the poor rates of patient response.^34^ Significantly, both immunotherapies and conventional chemotherapy are non-specific cancer treatments that frequently eradicate tumor cells while causing damage to numerous healthy cells, resulting in unwanted and occasionally lethal side effects. Efforts have been made for a considerable time to develop drug delivery systems that can target tumors, accumulate in tumor tissues, selectively identify and enter tumor cells, and precisely reach particular locations inside the cells.^35^ Several types of nanoparticles such as lipid-based polymeric nanoparticles, nanoparticles, and inorganic nanoparticles have been created to specifically transport therapeutic nucleic acids, chemotherapeutic drugs, or immunotherapeutic medicines to tumors. Advancements in nanotechnology, along with our increasing understanding of cancer biology and nano-bio interactions, have resulted in the creation of a range of nanocarriers. These nanocarriers could enhance the effectiveness of therapeutic drugs while reducing the unintended harm to healthy tissues. This is achieved by specifically targeting tumor tissue, cells, and organelles.^36^ The use of nano-medicine in clinical settings raises safety concerns, highlighting the need for a more comprehensive understanding of the essential properties that impact the interaction between nanoparticles and tissues and organs.^37^ Targeted nano-therapy has demonstrated superior efficacy and reduced toxicity compared to traditional anticancer treatments, due to its improved permeability, retention, and minimal side effects. It prolongs the duration for which nano-sized medications remain in the bloodstream and changes how they are distributed throughout the body, leading to the selective buildup of nanoparticles in tumor tissues.^38^ The prolonged plasma half-life of nanoparticles occurs when their size is above the threshold for renal excretion, hence impeding their elimination. The selective buildup of nanoparticles in tumor tissues leads to a greater concentration of the nano-sized medicine in these tissues than in the plasma or other organs. This accumulation is dependent on time and can be replicated in tumors of various sizes.^39^ This process leads to extended therapeutic effects, as well as enhanced targeting when the pharmacological actions and plasma concentration work together synergistically.^40,41^ Various therapeutic nanoparticle platforms including albumin nanoparticles (NPs), polymeric micelles, and liposomes have received approval for the treatment of cancer.^42^ Each entity possesses distinct attributes that confer advantages in particular applications, albeit with inherent limits in others.^43^ Chemotherapy has significantly increased the lifespan of countless cancer patients. Nevertheless, the extension of lifespan comes with a trade-off in terms of the deterioration of quality of life. Patients are susceptible to experiencing both immediate and long-lasting adverse effects including nausea and vomiting, chemotherapy-induced peripheral neuropathy, chemotherapy-induced alopecia (hair loss), cardiotoxicity, chemo brain, infertility, and diarrhea because of chemotherapy. At present, several of these negative consequences caused by chemotherapy are addressed by supportive care and licensed treatments, but the remaining side effects cannot be avoided. Therefore, chemotherapeutic medications, which have unavoidable side effects, are only given when their therapeutic benefits are greater than their toxic effects, thus greatly reducing the effectiveness of chemotherapy in treating cancer.^44^

## 2. Hyperthermia for cancer treatment

There has been a resurgence of interest in hyperthermia (HT) as a cancer therapy since Dr William Cooley made the initial observations in the 1890s that infections in cancer patients are connected to tumor regression and that injection of cocktails of attenuated bacterial cultures induces fever and a significant anti-tumor effect.^45^ Like surgical tumor removal, heat has frequently been used to ablate cancer cells. We call this thermo-ablation, a technique that uses extremely high heat to cause irreversible coagulation of proteins and other biological components as well as cell death. Hyperthermia is defined as treatment with increasing temperatures between 39 °C and 45 °C to induce cell death and increase intracellular oxidative stress levels.^46^ A temperature higher than 50 °C is referred to as thermo-ablation.^47^ It is important to emphasize that the effects of a temperature increase depend on the length of HT, the type of tissue, the temperature uniformity in the tissue, and the treatment environment. Necrosis and apoptosis of cancer cells can induce cell death even with temperature regimes as low as 42 °C maintained for longer than 1 hour.^48^ Electromagnetic radiation (such as laser, microwave, and radiofrequency) and high-intensity focused ultrasound are two methods utilized in modern practice to produce HT.^49^

There are three types of HT: whole body, regional, and local HT, depending on how much the body is exposed to heat.^50^ Whole-body hyperthermia (HT) can be achieved *via* thermal chambers and hot water blankets; in whole-body HTT, the temperature rises uniformly.^51^ Depending on the type of cancer being treated, regional heat therapy (HTT) is a procedure that involves heating large sections of tissues such as organs, limbs, or body cavities.^49^ In regional hyperthermia (HT), a heated solution of anticancer drugs is perfused into the peritoneum, and a portion of the patient's blood is extracted and warmed ex vivo to perfuse a tumor-bearing limb.^36^ These methods are technically difficult procedures to repeat with consistent heating settings, and they are not tumor-specific.^52^ The third type is local HT, which is tumor-specific. Localized HTT is employed for the treatment of cancer that is confined to a particular area. The application of this treatment can be achieved through external, intraluminal, and interstitial methods. Luminal and interstitial HT techniques utilize specific probes and applicators placed close to the tumor to achieve relatively uniform heating of the tumor.^49^ While HT is a treatment modality that holds great promise for cancer therapy, the methods of attaining, maintaining, monitoring, and modeling it suffer from many inadequacies. Therefore, there remains a continuing need for newer methods of generating HT.

### 2.1 Types of nanoparticle-based hyperthermia for cancer therapy

Nanoparticles are defined as materials with a diameter in the range of 1–100 nm. They display unique properties such as a larger surface area-to-volume ratio of nanoparticles that allows elemental metal atoms at the surface to have the greatest potential for interaction with surrounding molecules, nominating them for a wide range of applications in various fields of biomedicine such as nanobiotechnology, drug delivery, biosensing, and tissue engineering.^53^ Compared to larger materials, nanoparticles can move around more freely within the human body because of their small size. Furthermore, because of their high surface energy, nanomedicine's distinct mechanical, chemical, electrical, magnetic, biological, and structural qualities highlight the field's bright future in cancer research.^54^

The properties of nanoparticles mainly depend on their size, shape, and surface characteristics.^55^ For example, they have been found to have better optical and electrical qualities, greater magnetization, and higher reactivity than their bulk counterparts.^56^ Various materials have been used as precursors to produce nanoparticles of various sizes. Some of the most commonly used include metals, such as gold, silver, and platinum; metal oxides, such as titanium dioxide, iron oxide, and zinc oxide; and semiconductors, such as silicon, cadmium selenide, and zinc sulfide.^57,58^ Carbon-based compounds are among the other substances that have been used to create nanoparticles such as graphenes and carbon nanotubes.^59^ It is possible to coat nanoparticles with targeting molecules such as tumor-homing peptides and antibodies that allow docking to cancer-specific antigens to achieve even greater tumor accumulation and specificity, thereby reducing collateral thermal damage to adjacent normal tissues.^60^ From a mechanistic standpoint, targeted delivery of nanoparticles to tumors reduces the side effects of treatment.^61^

Nanoparticles are thought to be used for the production of hyperthermia treatment of cancers.^62^ It is possible to construct nanoscale transducers that specifically absorb tuned electromagnetic radiation and efficiently convert it to heat, which is then coupled and communicated to the tissues that the nanoparticles are contained in.^63^ The most common energy sources used for generating heat using nanoparticles are light, alternating magnetic fields, ultrasounds, and the modulation of internal factors, such as pH, temperature, redox potential, and enzymes.^47,64^ There are four main types of NP-mediated HTT depending on the external stimuli that can cause solid tumors to heat up without harming the surrounding tissues, namely, magnetically induced hyperthermia (MIH), photo thermal-induced hyperthermia (PIH), radiofrequency-induced hyperthermia (RIH), and ultrasound-induced hyperthermia (UIH), all of which exhibit exceptional potentials for cancer treatment.^64^

First, metallic nanoparticles, being excellent conductors of heat, efficiently transmit heat generated within them to adjacent tissues. Second, when administered intravenously, they can accumulate preferentially in tumors *via* the enhanced permeability and retention (EPR) effect, wherein particles smaller in size than a couple of hundred nanometers passively extravasate from leaky, chaotic and immature tumor blood vessels *via* large fenestrations (60–400 nm) in their vascular lining and are inefficiently cleared by an underdeveloped lymphatic drainage system.^65,66^

These particles are identified as foreign by circulating macrophages and resident reticuloendothelial cells in the liver and spleen, which then swallow them and remove them from the bloodstream. Reducing the size of the particles to below 5.5 nm has been shown to aid renal clearance and avoid reticuloendothelial capture.^67^ NPs are thought to be the best radio sensitizers for radiation therapy because of their high X-ray absorption and special physicochemical characteristics. They have demonstrated diagnostic and prognostic promise in cancer, revealing tumor location and disease stage^68^ and providing information regarding the efficacy of treatment.^69^ These nanoparticles can also carry anticancer therapeutic agents, which can be delivered in precise concentrations.

### 2.2 Magnetic nanoparticles

The magnetization of magnetic nanoparticles can vary arbitrarily and quickly in response to temperature variations, which causes the magnetization to be measured as zero (super paramagnetic) in the absence of an external magnetic field.^47,70–72^ Nevertheless, these super paramagnetic nanoparticles exhibit substantially higher magnetic susceptibility and behave like a single-domain paramagnet in the presence of a magnetic field. These super paramagnetic nanoparticles can be activated resonantly to produce heat when they are subsequently inserted into a tuned AMF. Several theories explain how super-paramagnetic nanoparticles convert AMF energy into heat.^47,73^ While AMF can generate heat in nanoparticles in tissues, non-magnetic material in tissues can also get heated unintentionally. When nanoparticles are carefully sized and shaped, as well as when the applied AMF frequency and amplitude are tuned, only the nanoparticle can be resonantly excited, with little to no off-target effects in normal tissues. The term “specific absorption rate” (SAR) refers to how well a nanoparticle converts absorbed AMF energy into heat. In biological systems, the preservation of SAR values may be impacted by the intracellular aggregation and destruction of nanoparticles. Super paramagnetic nanoparticles are typically less than 20 nm in size and resonant in magnetic fields between 10 kHz and 10 MHz, which allows them to easily enter soft tissues and bones.

One noteworthy characteristic of magnetic hyperthermia is that the AMF fields are usually focused on the body, instead of just the tumor. This implies that while the tumor is heated inside the AMF, untargeted particles that are present in healthy organs may also heat up. This makes using intravenous nanoparticle therapy to treat tumors near the liver or spleen difficult. An alternative approach is to inject the particles directly into the tumor to overcome this limitation of collateral heating of off-target tissue that has accumulated nanoparticles.^47^ Nonetheless, direct injection allows administering sufficient quantities of nanoparticles into the tumor to generate hyperthermia.

Żuk *et al.*, (2022) created iron oxide nanoparticles intended for use in radionuclide therapy and magnetic hyperthermia as part of a multimodal treatment for human epidermal growth factor receptor 2 (HER2-positive cancer). The gold-198 layer was applied to the magnetic core (Fe_3_O_4_) to produce core–shell nanoparticles. Subsequently, these were altered using a monoclonal antibody called trastuzumab and a bifunctional polyethylene glycol (PEG) linker to specifically target HER2-positive breast and nipple cancer cells. The synthesized nanoparticles demonstrated strong selective binding and internalization capabilities towards the SKOV-3 (HER2 positive) cancer cell line and demonstrated effective heat mediation in an alternating magnetic field.^74^

Salvanou *et al.* (2024) assessed the potential of iron oxide nanoparticles functionalized with bevacizumab, a monoclonal antibody, and doxorubicin, a chemotherapeutic agent, to act as a nano radiopharmaceutical agent against an aggressive triple-negative breast cancer cell line (4T1). An extra therapeutic benefit was obtained by direct radiolabeling with the medicinal isotope lutetium-177 (177Lu). High tumor accumulation and retention were seen at the tumor site following the intratumoral injection of functionalized radio-nanoconjugates. The produced nanoparticles have been identified as promising agents for Nano brachytherapy against breast cancer, as evidenced by the therapeutic efficacy research that showed improved retention of tumor growth.^75^

Kossatz *et al.* (2015) functionalized the super paramagnetic iron oxide nanoparticles (MF66) electrostatically with either doxorubicin (DOX; MF66-DOX), or Nucant multivalent pseudo peptide (N6L; MF66-N6L), or both (MF66-N6LDOX). The effectiveness of treatment was evaluated in breast adenocarcinoma cell line MDA-MB-231 tumor-bearing female athymic nude mice. They discovered that in the alternating magnetic field, every variation of the nanoparticle exhibited a great heating potential. When combined with heat, MF66-DOX and MF66-N6LDOX were more lethal to breast cancer cells. They found that intratumoral injection of the nanoparticles significantly inhibited the growth of the tumors *in vivo*. The residual tumor tissue's capacity to proliferate was markedly inhibited. Combining MF66 functionalized with N6L and DOX with magnetic hyperthermia may significantly improve the therapeutic effects of breast cancer magnetic hyperthermia.^76^

### 2.3 Gold nanoparticles

Gold nanoparticles (AuNPs) are stable nanomaterials that are used for the preparation of nanostructures with various shapes.^77^ Consequently, it has been possible to create photo thermally activated gold nanoparticles with a gold surface layer and a hollow inner core composed of either gold or silica. The near-infrared (NIR) portion of the spectrum, which falls between 700 and 850 nm, is the wavelength of greatest relevance for therapeutic applications since it is the optical window where light penetrates biological tissues the deepest. It is possible to adjust the resonance wavelength of gold nanoparticles to match the incident light by changing their size, shape, and composition.^78^ The recent advancement in technology has led to accurate surface coating of Au NPs with specific particle shapes and sizes. These specificities of gold nanomaterials make it a safer and specific anticancer and drug delivery agents.^79^

Moreover, Artificial Intelligence revealed that gold nanoparticles can adjust their optical densities, light absorbency, and wavelengths. Therefore, adjusting the ideal wavelength with nanoparticle size may allow a higher amount of light absorbance within the nanoparticle itself, leading to enhanced efficacy of gold nanoparticles against cancer cells.^80^ In a recent study, gold NPs increase cisplatin delivery and potentiate chemotherapy.^81,82^ Moreover, AuNPs can directly conjugate with several molecules including antibodies, nucleic acids, proteins, enzymes, fluorescent dyes, and drugs, which enhance their applications in the medical field.^83,84^ Gold nanospheres can also easily be conjugated with several imaging reporters and can carry genes, drug payloads, and other chemotherapeutic agents for theranostic applications. Gold nanoparticles usually passively accumulate in tumors and exert specific pharmaceutical effects with active targeting ligands, such as Apts, antibodies, and peptides to the required targets.^85^

The gold nanoparticles (GNPs) may also be used to enhance the anticancer efficacy of Fluorouracil (5-FU) and reduce its side effects. 5-FU can be loaded to GNPs using thiol-containing ligands, thioglycolic acid (TGA), and glutathione (GSH) as 5-FU/GSH-GNPs. Further study revealed that the release of 5-FU from GNPs was slow and induced apoptosis in colorectal cancer cells. Overall, 5-FU/GSH-GNPs showed two-fold higher anticancer efficacies than those with free 5-FU.^86^ The Center of Cancer Nanotechnology at Stanford University developed a technique where gold nanoparticles are specifically allowed to bind the colorectal cancer (CRC) cells. Subsequently, light from a colonoscope is allowed to shine, and the gold nanoparticle-bound cancer cells stand out from the normal cells that can be removed easily.^87^

Żelechowska-Matysiak *et al.* (2023) developed a multimodal radio-bioconjugate that contains a chemotherapeutic agent (doxorubicin, DOX), a β-emitter (^198^Au), and a guiding vector (trastuzumab, Tmab) for targeted treatment of cancers overexpressing HER2 receptors. To achieve this goal, radioactive gold nanoparticles (^198^AuNPs) were synthesized and coated with a poly(ethylene glycol) (PEG) linker conjugated to DOX and monoclonal antibody (Tmab). *In vitro* experiments demonstrated a high affinity of the radio-bioconjugate to HER2 receptors and cell internalization. Cytotoxicity revealed a marked reduction in SKOV-3 cell viability. Analysis using flow cytometry revealed that DOX-198AuNPs-Tmab mostly caused late apoptosis and cell cycle arrest in the G2/M phase. Finally, *in vivo* therapeutic efficacy studies on the same animal model demonstrated a significant tumor growth arrest up to 28 days following a single intratumoral injection of 10 MBq. Therefore, the proposed multimodal radio-bioconjugate shows great potential for the local treatment of HER2+ cancers.^88^

### 2.4 Carbon nanotubes

Carbon nanotubes (CNTs), allotropes of carbon cylindrical in shape, have rolled graphene sheets that are fewer than 1 μm in diameter and a few nanometers in length.^89,90^ The chemical and physical characteristics of carbon nanotubes (CNTs), including their large surface area, needle-like shape, thermal conductivity, and chemical stability, make them suitable for application in various sectors including gene therapy, sensors, immunotherapy, diagnostics, and drug delivery systems.^91,92^ While multiwalled carbon nanotubes (MWCNTs) are made up of many such tubes concentrically stacked within one another, single-walled carbon nanotubes (SWCNTs) only have one roll of the graphene sheet. A wide range of incoming energies including visible light, near-infrared light, and even radiofrequency waves are absorbed by CNTs. Electronic transitions within the nanoparticle are triggered by electromagnetic waves, and the relaxation that ensues causes the vibrational modes within the carbon lattice to be enhanced. Changes in length and diameter, the number of concentric tubes and dopants inside or outside the cylinder, and the arrangement of carbon atoms in the cylinder wall for strong thermal conductivity are examples of tunable features.

**Fig. 1:**
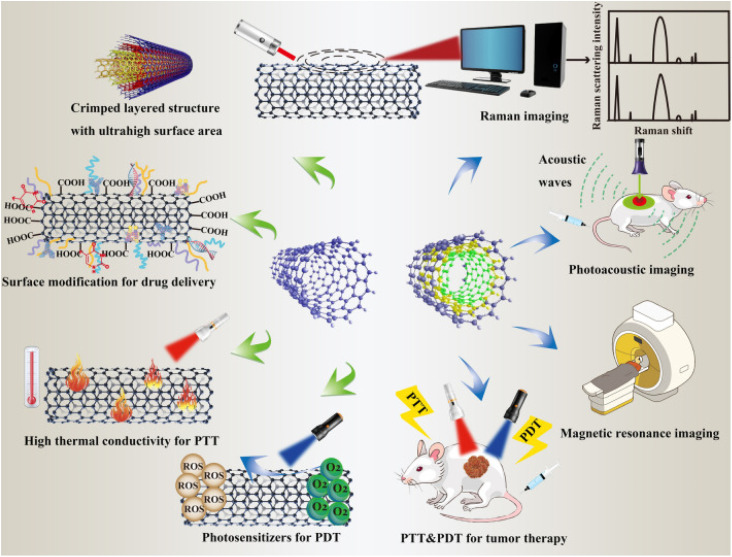
Representation of the versatile functionalization of CNTs in cancer therapy (adapted with permission from ref. 95, Biomed Central lab, (2021)).

These CNTs' customizable thermal, electrical, and spectroscopic characteristics make them extremely adaptable conduits for various biological applications such as the generation of HT.^89,93^ Mice with head and neck squamous cell carcinoma xenografts were directly injected with SWCNTs, and the tumors were completely eradicated after three minutes of NIR laser illumination. However, an Eschar developed on the skin as a result of heat injury.^78^ After two months, the nanotubes were mostly excreted from the tumors by the kidneys and liver. When implanted intratumorally into kidney cancer xenografts in mice, MWCNTs, with their higher absorption cross-sections than SWCNTs, demonstrated strong anti-tumor activity before the tumors were exposed to brief (30 seconds) bursts of low-power (3 W cm^−2^) laser light.^94^ The extent of tumor regression was dose-dependent and durable long-term tumor control was observed with higher doses of MWCNTs. Fig. 1 displays the employment of CNTs in cancer therapy.

Furthermore, these MWCNTs may be susceptible to some off-target toxicity if they are activated by tailored radiofrequency waves. One ongoing worry regarding carbon nanotubes is the potential for mice's mesothelial and pleural linings to become exposed to them, leading to the development of granulomas similar to asbestos-related mesotheliomas.^96^ Numerous studies revealed that multiple approaches to the delivery of anticancer medicines have been devised using carbon nanotubes. Using single-walled carbon nanotubes coupled with a synthetic polyampholyte, paclitaxel was delivered to Caco-2 cells. The anticancer effects of paclitaxel-SWCNT-treated Caco-2 and HT-29 cells were higher than those of paclitaxel alone.^97^ Eudragit®-irinotecan-loaded CNTs have shown similar kinds of results.^98^ By heating the cell membrane, oxaliplatin and mitomycin C-coated CNTs were able to localize and transport drugs much more effectively in cancerous colon cell lines when exposed to infrared light.^99^

Moon *et al.* (2009) showed that following intratumoral injection of single-walled carbon nanotubes (SWCNTs) into mice bearing flank xenografts of human mouth carcinoma cells and near-infrared irradiation (NIR), the tumors were eliminated. The surrounding normal tissue was not damaged by the treatment; however, even without SWCNTs, the irradiation process caused the heated region to burn significantly. Remarkably, throughout several months of follow-up, no signs of tumor recurrence or evident side effects of treatment were noticed.^100^

Moreover, the CD133 receptor seems to be a marker for cancer stem cells (CSCs) in glioblastomas and other brain tumors, and it is related to malignancy, tumor recurrence, and poor prognosis. After radiation therapy, CD133+ subpopulations in glioblastoma are enriched, resistant to chemotherapy and radiation, and may be in charge of tumor recurrence after treatment.^101^ Treatment strategies based on targeting this subpopulation may prevent the development of therapy resistance. Thus, Wang *et al.* conjugated a monoclonal antibody directed against CD133 to Multi-Walled Carbon Nanotubes (MWCNTs).^102^ They observed specific absorbing of these targeted MWCNTs in primary clinical isolates of glioblastoma that expressed CD133, but not in cells that did not. Before injection into mice, CD133-expressing glioblastoma cells were pre-treated with targeted MWCNTs to assess the treatment's *in vivo* effectiveness. The cells took up the MWCNTs, and xenograft growth was eliminated after NIR exposure. This important study demonstrated the potential for CNTs to treat glioblastomas and other currently untreatable cancers.^102^

Farzin *et al.*^103^ proposed a simple and label-free voltammetric immunosensor for the rapid detection of prostate-specific antigens (PSAs). Till now, several immunosensing technologies such as fluorescence immunosensors,^104^ electrochemical immunosensors,^103^ surface-enhanced Raman scattering-based immunoassays,^105^ and surface plasmon resonance (SPR) immunosensors are used for PSA detection. Farzin *et al.*^103^ Proposed an immunosensor based on the multi-walled carbon nanotube (MWCNT)/l-histidine-immobilized reduced graphene oxide (His-rGO) for attaching a thionine redox indicator and an anti-PSA antibody (Ab). MWCNTs play a crucial role in the facilitation of electron transfer between thionine and the glassy carbon electrode and the enhancement of electrical conductivity. In another study, Soares *et al.* also developed an MWCNT-based immunosensor for the detection of the pancreatic biomarker CA19-9.^106^ The actual patient blood serum samples with various CA19-9 concentrations have been analyzed using the sensor. The findings demonstrated precise detections despite analyte interference from bodily fluids. Consequently, it can be considered a potent, efficient, straightforward, and precise method of identifying pancreatic cancer in its early stages.

### 2.5 Metal–organic frameworks (MOFs)

Therapeutic drugs can partially attack tumor sites; moreover, distinguishing cancer cells from normal cells without damage to the healthy tissues is not easily achievable. The use of MOFs to an acceptable level compensates for such an inability.^107^ Metal–organic frameworks (MOFs), which are novel porous materials, are manufactured using metal nodes and organic linkers.^108–112^ They offer a tunable design and a network structure with controlled chemical functionality, high crystallinity, and good porosity. Due to their unique structures and properties, MOFs have been applied in gas storage and separations,^113^ catalysis,^114^ energy, and sensing.^115^ The MOF-based biosensors have currently been applied for the detection of various targets such as heavy metal ions,^116^ hazard molecules,^117^ and living cancer cells.^118^ Most of the biosensors have been designed either for the detection of cancer markers,^119^ or small biomolecules released from cancer cells for early diagnosis.^116,120^ MOFs are promising platforms for drug delivery because of their porosity, tunable design, and low toxicity.^121–123^ Furthermore, MOFs were also good delivery platforms for the bio macromolecules, and the loading strategies of bio macromolecules can be categorized into four ways: (i) the bio macromolecule was adsorbed on the surface of MOFs due to the physical absorption;^124,125^ (ii) the bio macromolecule was conjugated on the surface *via* chemical coupling with the organic bridging ligands;^126^ (iii) the bio macromolecule was infiltrated into the pore taking advantage of the mesoporous nanostructure;^127^ and (iv) the bio macromolecule was encapsulated within the MOF networks during the self-assembly reaction of the mixed solution containing metal cations, organic bridging ligands and the bio macromolecules.^128^

MOFs are widely used as tailorable theranostic platforms for both cancer diagnosis and cancer treatment, including monomodal therapeutics such as photodynamic therapy (PDT), photo thermal therapy (PTT), chemotherapy, radiography, and immunotherapy.^107^ The strategy is based on modification of MOFs to make them photosensitizers working efficiently under a specified laser irradiation wavelength, resulting in hybrid photosensitizing agents inducing cancer cell apoptosis.^129,130^ MOFs are highly porous biocompatible tailorable hybrid structures with therapeutic effects on cancerous cells as a result of their ability to encapsulate cargos (drugs, proteins, genes, *etc.*).^107^ Subsequently, combined MOFs with additional electrochemically active components have emerged as a successful tactic for utilizing MOF-based cytosensors. In one such study, the MOF was employed to create bimetallic TbFe-MOFs, which were then used to anchor carbohydrate antigen 125 aptamers for the identification of live Michigan Cancer Foundation-7 (MCF-7) cells.^131^ Similarly, the bimetallic ZrHf-MOF doped with carbon dots was used in the identification of human epidermal growth factor receptor-2 (HER2) and HER2 expressed in MCF-7 cells.^118^ Later, combinations of MOFs with different types of nanomaterials such as metal oxides, metal nanoparticles, and carbon-based nanomaterials were used to synthesize newer forms of multifunctional composites.^132^ A chlorin-based nanoscale metal–organic framework was used for photodynamic therapy of colon cancers using mouse models and found that these have great potential for clinical translation.^133^ In a recent study, a zirconium-based metal–organic framework (MOF), PCN-223, has been synthesized and used as a potential vehicle for 5FU for rectal delivery in the diagnosis of CRC.^134^

## 3. Nanoparticle-based drug delivery systems and cancer therapy

**Fig. 2:**
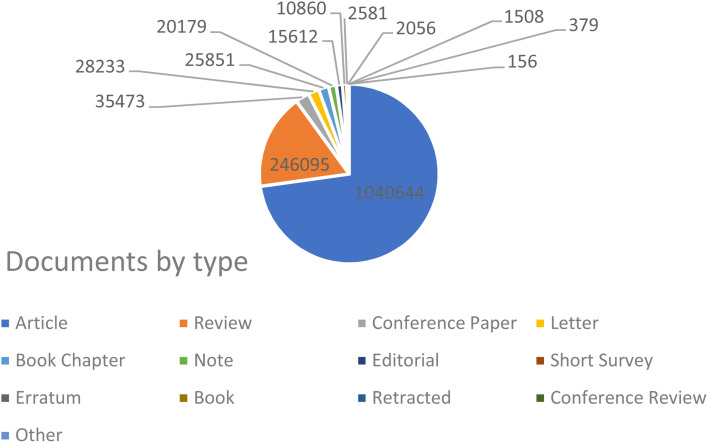
Representation of the progressively increasing number of publications on the keyword “cancer treatment” during the period of 2000–2024 (Source: Scopus database, accessed 07112024).

**Fig. 3:**
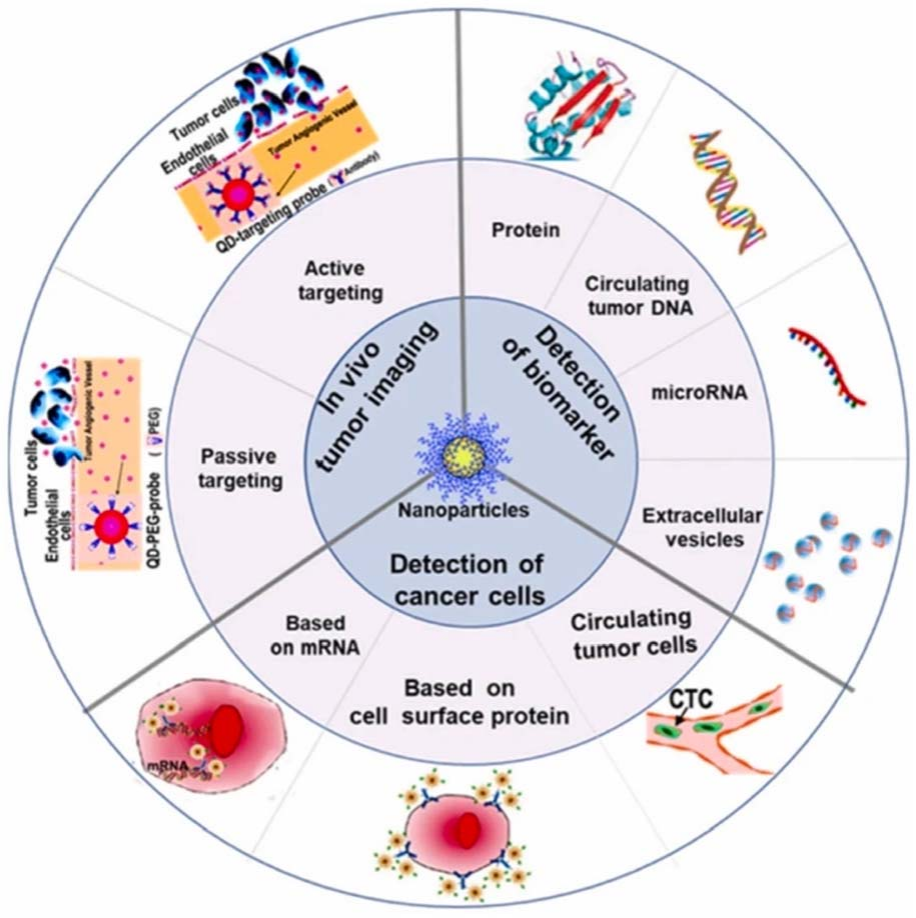
Schematic of nanotechnology applications towards cancer diagnosis (adapted with permission from ref. 143. Copyright, Springer (2019)).

Drug-delivery systems have been a prominent area of multidisciplinary research for almost two decades, resulting in significant advancements in treating many diseases.^135,136^ Drug delivery to areas of the body that were previously inaccessible, such as traversing the blood–brain barrier to treat neurological disorders, has been made possible through the application of nanotechnology.^137^ Nanotechnology has been thoroughly researched and utilized for cancer therapy due to the ability of nanoparticles to serve as an effective drug delivery mechanism. Nanoparticle-based medication delivery offers enhanced stability, biocompatibility, improved permeability, and retention impact, as well as targeted dispersion compared to conventional drug delivery methods. Fig. 2 represents the increasing number of publications towards nanomaterial applications in cancer treatment. Hybrid nanoparticles, which mix the features of several nanoparticles, have advanced drug-carrier systems to a higher degree. Nanoparticle drug delivery methods have been demonstrated to help overcome medication resistance in cancer treatment. Cancer medication resistance mechanisms involve increased production of drug efflux transporters, impaired apoptotic pathways, and low oxygen levels. Nanoparticles that target these processes can enhance the reversal of multidrug resistance. Moreover, nanoparticles are being progressively designed to target newly discovered tumor drug resistance pathways. Scientists have begun studying the impact of nanoparticles in immunotherapy, which is increasingly significant in cancer treatment.^69^ Targeting of tumors by nanoparticles is determined by their structure and composition. A prevalent strategy involves the affixed surface of nanoparticles with targeting molecules capable of identifying and binding to receptors present on tumor cells.^138^ Such a mechanism enables the nanoparticle to accumulate specifically within the tumor, thereby augmenting its effectiveness while reducing harm to viable cells.^139^ Furthermore, certain nanoparticles have the capability to be modified, so that they discharge their therapeutic payload in reaction to particular stimuli, including fluctuations in pH or temperature.^140^ Utilizing metal nanoparticles in medication delivery systems allows for the direct targeting of the damaged organ, which, in turn, lowers the occurrence of unwanted side effects.^141^ In addition, when magnetic nanoparticles are coated with silica, it results in the creation of bifunctional nanostructures that have significant potential for use in biomedicine and pharmacy. Research has demonstrated that magnetic mesoporous silica nanoparticles (MMSNs) can be used as a contrast agent in MRI analysis. Furthermore, these nanocomposites have been used as an optimal framework for the concurrent administration of chemotherapy and hyperthermia treatment to augment the therapeutic effectiveness of conventional chemotherapy.^142^Fig. 3 reflects the nanotechnology consideration towards cancer therapy.

### 3.1 Metallic nanoparticles as a drug delivery system

**Fig. 4:**
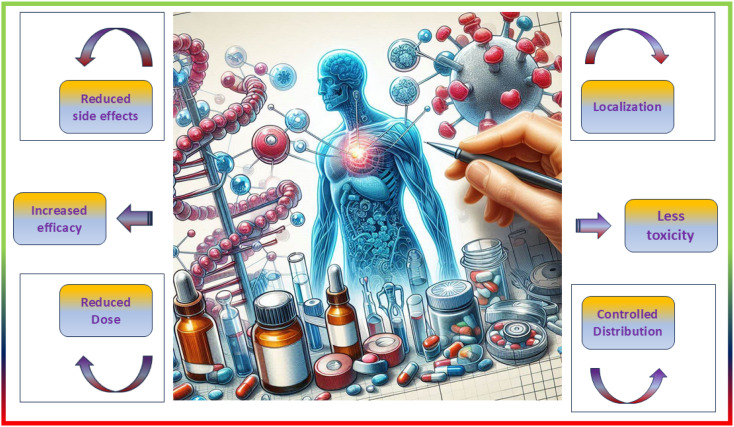
Benefits associated with targeted drug delivery systems.

Drug delivery systems (DDS) are an area of medical sciences that are perpetually evolving and represent one of the most promising applications of human health care. Despite substantial advancements in the domain of drug delivery systems (DDS), formulation scientists continue to face a critical obstacle in the form of developing a suitable vehicle that is both efficient and offers the highest possible benefit-to-risk ratio for drug delivery to the body.^141^ The use of MNPs as carriers for drugs to treat tumor cells has been rapidly improving in recent years.^144^ Metal nanoparticles (MNPs) have gained considerable interest for their ability to enhance drug effectiveness in medicine by targeting specific sites, reducing drug resistance, and improving drug delivery efficiency. Metal nanoparticles (MNPs) are highly intriguing due to their optical characteristics, particularly surface plasmon resonance (SPR), which allows for manipulation of the optical field. This feature makes MNPs promising contenders for biomedical applications. The compact dimensions of MNPs enable them to penetrate through biological or physiological membranes that are often impenetrable to other large molecules.^145^ Using metallic nanoparticles in drug delivery systems has significant advantages such as improved stability and extended half-life of the drug carrier in circulation, essential biodistribution, and passive or active targeting to the intended location.^141^ To modify the pharmacokinetic properties of MNPs, it is possible to adjust the surface to that effect. For instance, the circulation duration of MNPs within the organism can be prolonged by coating their surface with polyethylene glycol (PEG), which inhibits non-specific uptake by the mononuclear phagocyte system. In recent years, the utilization of MNPs as drug delivery vehicles for the treatment of tumor cells has advanced significantly.^144^ An important part of MNP surface chemistry is the attachment of targeting ligands—antigens, peptides, or nucleic acid sequences—to certain disease organs or tissues.^146,147^ The main reasons for obtaining MNPs are their properties to increase the water solubility of hydrophobic pharmacological compounds, to slow down or stop the rapid renal drug excretion, and to increase the amount of time that medicines spend in circulation. Multifunctional nanoparticles exhibit enhanced capabilities compared to traditional nanoparticles. They have the potential to accomplish several goals in tandem, such as the delivery of various bioactives in conjunction with imaging agents, the decoration of surfaces with ligands to target specific areas, and the provision of cancer diagnostics and therapies all at once.^141^ Drug delivery aims to direct therapeutic agents to specific sites, reduce unwanted effects on healthy tissues, and regulate drug release to prevent overdosing or underdosing.^148^ Successful medication delivery *via* MNPs relies on two crucial factors: (i) the design of MNPs to facilitate gradual and continuous drug release and (ii) the capability of MNPs to transport therapeutic medicines to specific target areas while minimizing the impact on surrounding healthy cells.^149^ These variables could be easily attained using active and passive targeting. Passive targeting occurs because of specific alterations in the cancer blood vessels. Due to rapid tumor growth, blood arteries and junctions may become misshapen, leading to leakage and instability. Because of their distinctive size, MNPs can pass through these porous junctions, leading to enhanced accumulation at the tumor site, making them largely used for cancer treatment over time.^150^ In contrast, active targeting involves conjugating MNPs with various ligands that bind to particular cell surface receptors; this leads to the delivery of the drug load to the target region.^151^ Clinically validated metal nanoparticle-based nanomedicines have demonstrated better bioavailability and efficacy of drug delivery systems, as well as reduced adverse effects, through enhanced targeted delivery and cellular uptake. To make designed MNPs that are easily digested by various metabolic routes and that break down rapidly under specific physiological conditions, their sizes, morphologies, surface chemistry, and doping procedures have been carefully controlled.^152^ Metallic nanoparticles exhibit a multitude of features, which have significantly expanded the possibilities in nanotechnology, particularly in the development of targeted medication delivery systems^153^ (Fig. 4).

**Fig. 5:**
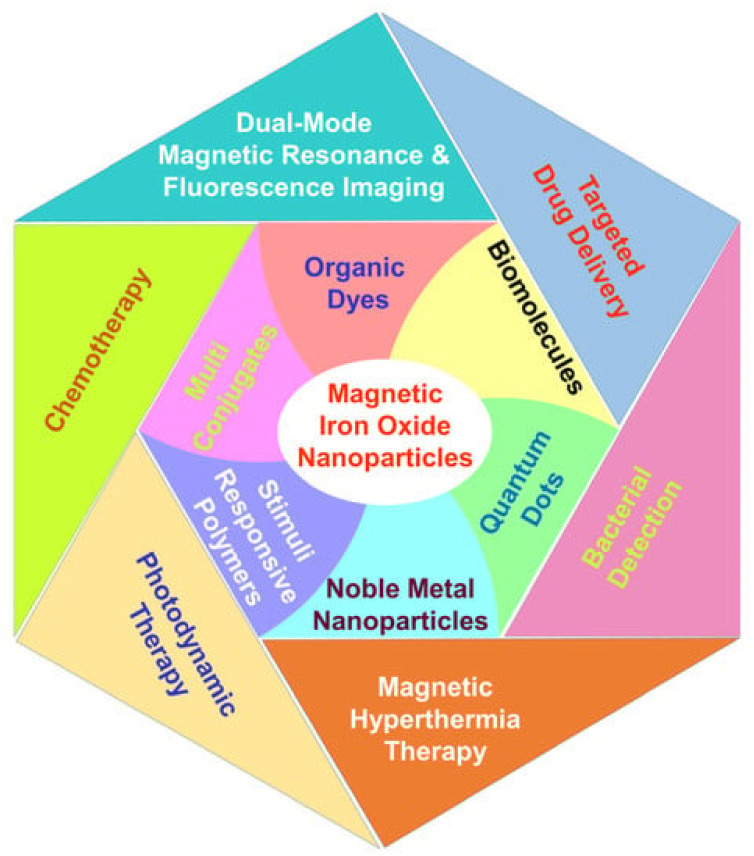
Multifunctional magnetite nanoparticles conjugated with various categories of organic and inorganic materials directed toward biomedical applications (adapted with permission from ref. 158. Copyright, MDPI publisher, (2022)).

MNPs are widely used as carriers for delivering different therapeutic agents such as antibodies, nucleic acids, chemotherapeutic medicines, peptides, and more. The majority of MNPs such as silver, gold, palladium, titanium, zinc, and copper nanoparticles exhibit improved and adjustable optical characteristics.^154,155^ In addition, the surface of these particles can be readily modified to attach targeted agents and active biomolecules using hydrogen bonding, covalent bonding, and electrostatic interactions. Furthermore, it is possible to conveniently load different medications to enhance the therapeutic effectiveness.^156^ MNPs are obtained due to their potential to boost the solubility of hydrophobic medicinal compounds in water, prolong the circulation time of medications in the bloodstream, and inhibit or eliminate rapid renal excretion of drugs.^157^ Multifunctional nanoparticles exhibit superior capabilities compared to conventional nanoparticles. They can synergistically achieve multiple objectives, such as delivering multiple bioactives along with imaging agents, targeting specific areas through surface ligand decoration, and simultaneously providing cancer therapeutics and diagnostics^141^ (Fig. 5).

Drug delivery aims to achieve three primary objectives: directing therapeutic agents to the specific site of action, reducing the negative impact of drugs on healthy tissues or organs, and regulating the release of drugs to prevent the traditional problem of overdosing or underdosing.^148^ MNPs offered a paradigm to accomplish these objectives. Therefore, the optimization of the coating on the surface of MNPs has been done to precisely regulate the amount of drug loaded, the distribution of the drug, and the release of the drug in the specific target area.^159^ Nanotechnology and Nano drugs, with their interdisciplinary and multipurpose nature, have facilitated diversification and improvements aimed to enhance the quality of life. Currently, the fundamental principles of nanotechnology as applied to medicine and the development of Nano drugs have not been thoroughly investigated. Hence, it is imperative to do a thorough study in the field of nanomedicines, particularly in drug delivery systems. The field of nanomedicines is continuously seeking novel and enhanced therapies for diseases, which must exhibit both high effectiveness and cost-efficiency. This places a significant burden on scientific research to identify these innovative treatments. An essential feature of any treatment is the capacity to specifically target the ailment without causing injury to any other healthy area of the body. In recent times, there has been a significant advancement in the use of metal nanoparticles as targeted drug delivery systems due to their ready availability, biocompatibility, and stability. These behaviors enable the medications to be encapsulated and delivered directly to the targeted areas, resulting in a greater biological effect. The advancement of metallic nanoparticles is swift and diverse, and their enhanced practical capabilities emphasize their effectiveness as innovative instruments for future drug delivery methods, particularly in the treatment of cancer, inflammation, diabetes, and antiviral therapy, concluded by Chandrakala *et al.*^141^

### 3.2 Core–shell structure of @mSiO_2_, @Cs

Therapeutic drug delivery and cancer imaging have made extensive use of nanotechnology-based systems.^160^ Inorganic nanoparticles have been widely used as delivery systems due to their exceptional physical and chemical characteristics.^161^ Inorganic nanoparticles are commonly utilized in drug delivery systems because of their distinctive physical and chemical characteristics including simple preparation/modification, excellent biocompatibility, and storage durability.^160^ Researchers are highly interested in constructing nanocomposite carriers with adjustable shape and homogeneous size due to the significant impact of nanoparticle size and shape on their biological uses.^161–163^ The widespread use of core–shell nanoparticles in biomedical imaging therapy and drug delivery has contributed to their rise to prominence in the past several decades. Particles with a core–shell composite structure have two or more parts organized in a certain way. In order to construct carriers with varying degrees of functionality, researchers often combine metal oxides, nonmetal oxides, and polymers.^164,165^ Magnetic nanoparticles (MNPs), specifically iron oxide nanoparticles (Fe_3_O_4_), have attracted considerable attention in the disciplines of pharmacy and biomedicine.^166^ Magnetic nanoparticles are utilized in novel drug delivery systems to provide controlled drug release by applying an external magnetic field to the specific location.^167^ Additionally, these systems have the potential to be utilized in thermotherapy applications for producing heat using an alternate magnetic field.^168^ Nevertheless, the unaltered magnetic nanoparticles (MNPs) exhibit inadequate biocompatibility and a low capacity for drug loading, rendering them inappropriate for use in biomedical applications. Applying a layer of mesoporous silica onto the surface of MNPs can prevent the clumping together of nanoparticles and improve their ability to interact with living organisms. Moreover, the mesoporous silica coating offers numerous benefits owing to its distinctive features such as a substantial surface area and a large pore volume, leading to a heightened effectiveness in drug-loading.^169^ Hence, magnetic mesoporous silica nanoparticles (MMSNs) have the potential to be employed in both chemotherapy and thermotherapy, making it a very efficient approach for cancer treatment.^170^ Furthermore, some studies have indicated that silica-coated magnetic nanoparticles have the potential to enhance contrast in MRI applications. Nevertheless, there are certain constraints associated with the utilization of MMSNs as vehicles for medication delivery. One of the most difficult problems in targeted medicine delivery systems is the premature release of cargo from the carrier before it reaches the intended place. This process leads to the destruction of healthy tissues and greatly reduces the effectiveness of chemotherapy. Grafting stimuli-responsive polymers onto the surface of the silica shell is a highly effective strategy for resolving this issue.^171^ Gatekeepers can effectively hinder or minimize the escape of drugs from the carrier before they reach the intended destination. Additionally, they can facilitate the release of drugs in response to internal stimuli such as pH, enzymes, and redox reactions, or external stimuli such as light, magnetic fields, and ultrasound at the target region.^171^ In addition, to enhance the regulation of drug release from the carrier, dual-stimuli-responsive systems were developed. These systems include pH/light, pH/magnetic field, and temperature/magnetic field responsive systems. Poly(*N*-isopropyl acrylamide) (PNIPAAm) is a very desirable temperature-sensitive polymer that can function as an efficient gatekeeper on the surface of MSNs.^170^

**Fig. 6:**
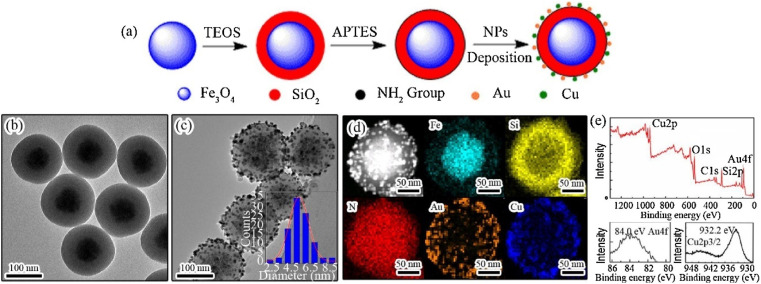
(a) Stepwise preparation process of Fe_3_O_4_@SiO_2_–Au/Cu magnetic nanocomposites. TEM images of (b) Fe_3_O_4_@SiO_2_ and (c) Fe_3_O_4_@SiO_2_–Au/Cu nanoparticles. The inset in (c) depicts the nominal size distribution of Au/Cu NPs over the silica surface, determined by calculating the diameters of around 200 particles in the TEM images. (d) High-angle annular dark-field STEM image of the Fe_3_O_4_@SiO_2_–Au/Cu nanocomposite, accompanied by the elemental mapping of Fe, Si, N, Au, and Cu and (e) XPS survey of the Fe_3_O_4_@SiO_2_–Au/Cu nanocomposite and high-resolution Au 4f and Cu 2p spectra (adapted with permission from ref. 172, Elsevier, (2018)).

**Fig. 7:**
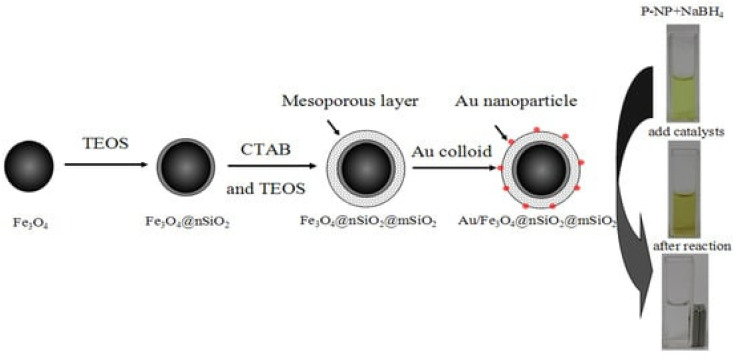
Representation of the preparation process of Au/m-SiO_2_/Fe_3_O_4_, which might be separated using a magnet (adapted with permission from ref. 177. Copyright MDPI publisher, (2013)).

A unique technique is proposed by Asgari *et al.*^169^ for encapsulating Fe_3_O_4_ nanoparticles with a mesoporous silica shell using a modified reverse micro emulsion system. In order to enhance the regulation of drug release in the produced nanocomposites, a temperature-responsive copolymer P (NIPAAm-*co*-AAc) was attached to the surface of the mesoporous silica shell using the radical polymerization technique. Subsequently, the produced nanocomposites were used to load 5-FU, a model anti-cancer chemotherapeutic medication. Following that, the drug release behavior of the nanocomposites that were created was examined at temperatures both below and beyond the lower critical solution temperature (LCST) of the polymeric shell. Additionally, the drug release characteristics of the produced nanocomposite were examined when exposed to an alternating magnetic field (AMF). The drug release mechanism from P(NIPAAm-*co*-AAc)@mSiO_2_@Fe_3_O_4_ nanocomposites was investigated utilizing several theoretical kinetics models.^170^ For use as a magnetic/temperature-responsive drug delivery vehicle, a type of nanocomposite with a core–shell–shell structure comprising Fe_3_O_4_ nanoparticles in the center, mesoporous silica in the middle shell, and the temperature-responsive P(NIPAAm-*co*-AAc) copolymer as the outer shell was successfully synthesized. The mSiO_2_@Fe_3_O_4_ nanoparticles were verified to have an average particle size of less than 100 nm and were coated with a polymer shell, as validated by FTIR and TEM investigations. Furthermore, the hyperthermia analysis results indicate that both mSiO_2_@Fe_3_O_4_ and P (NIPAAm-*co*-AAc)@mSiO_2_@Fe_3_O_4_ nanocomposites possess the capability to function as a heat source in magnetic hyperthermia applications. Additionally, the 5-FU carrier performance of P (NIPAAm-*co*-AAc)@ mSiO_2_@Fe_3_O_4_ nanocomposites was assessed. The efficacy of the outer polymeric shell as a temperature-responsive gatekeeper was demonstrated by the drug release profile observed at 37 °C and 45 °C. Additionally, a burst drug release was observed when the prepared nanocomposites were exposed to a safe alternating magnetic field utilized in magnetic hyperthermia therapy. The results obtained indicate that the P(NIPAAm-*co*-AAc)@ mSiO_2_@Fe_3_O_4_ nanocomposites that were prepared possess significant potential for utilization in advanced drug delivery applications as magnetic/temperature dual-responsive drug carriers^170^ (concluded by (Asgari *et al.*^169^)). CSs have gained scientific interest due to their promising characteristics, including biocompatibility, biodegradability, and bioactivity, surpassing other materials such as gold and silica.^173^ In addition, they demonstrate high drug loading efficiency and a prolonged drug release.^174^ Researchers conducted numerous research to incorporate these materials into biomedical applications such as bone regeneration and medication delivery systems.^175^ Lu *et al.* fabricated a magnetic iron oxide-CS mesoporous core–shell nanocomposite as a potential carrier for drug delivery purposes.^176^ The synthesis method was carried out employing ultrasound irradiation with two liquid-phase systems, while varying the amount of isooctane as an inert hydrophobic solvent.^175^Fig. 6 and 7 represent the process of fabrication of @mSiO_2_ core–shell structures.

### 3.3 Stimuli-responsive hydrogels for cancer treatment

Different definitions of hydrogels have been suggested through the years. Often, they are described as a water-swollen and cross-linked polymeric network produced by the reaction/conjugation of one or more monomers. However, more recently, hydrogels have been represented as three-dimensional networks which may absorb large amounts of water (from 10 to 20% up to thousands of times their dry weight) due to the presence of hydrophilic functional groups, which fill the space among macromolecules and show high affinity for biological fluids.^178^ Until today, hydrogels are routinely present in people using contact lenses, especially soft lenses based on silicon hydrogels. Moreover, hydrogels have been extensively used in biomedical applications regarding tissue engineering and wound healing.^179,180^ Advanced dressings based on hydrogels proved to be more effective in wound healing since they maintain a moist environment at the application site that avoids the spread of fluids to other healthy areas of the skin. Stimuli-responsive hydrogels are promising smart materials able to change conformation as a response to surrounding environment variations such as temperature, pH, light, ionic strength, and magnetic field. This type of hydrogel has gained special importance due to the possibility of manipulating the rheological behavior of the hydrogel according to the different tumor microenvironment conditions.^181^

**Fig. 8:**
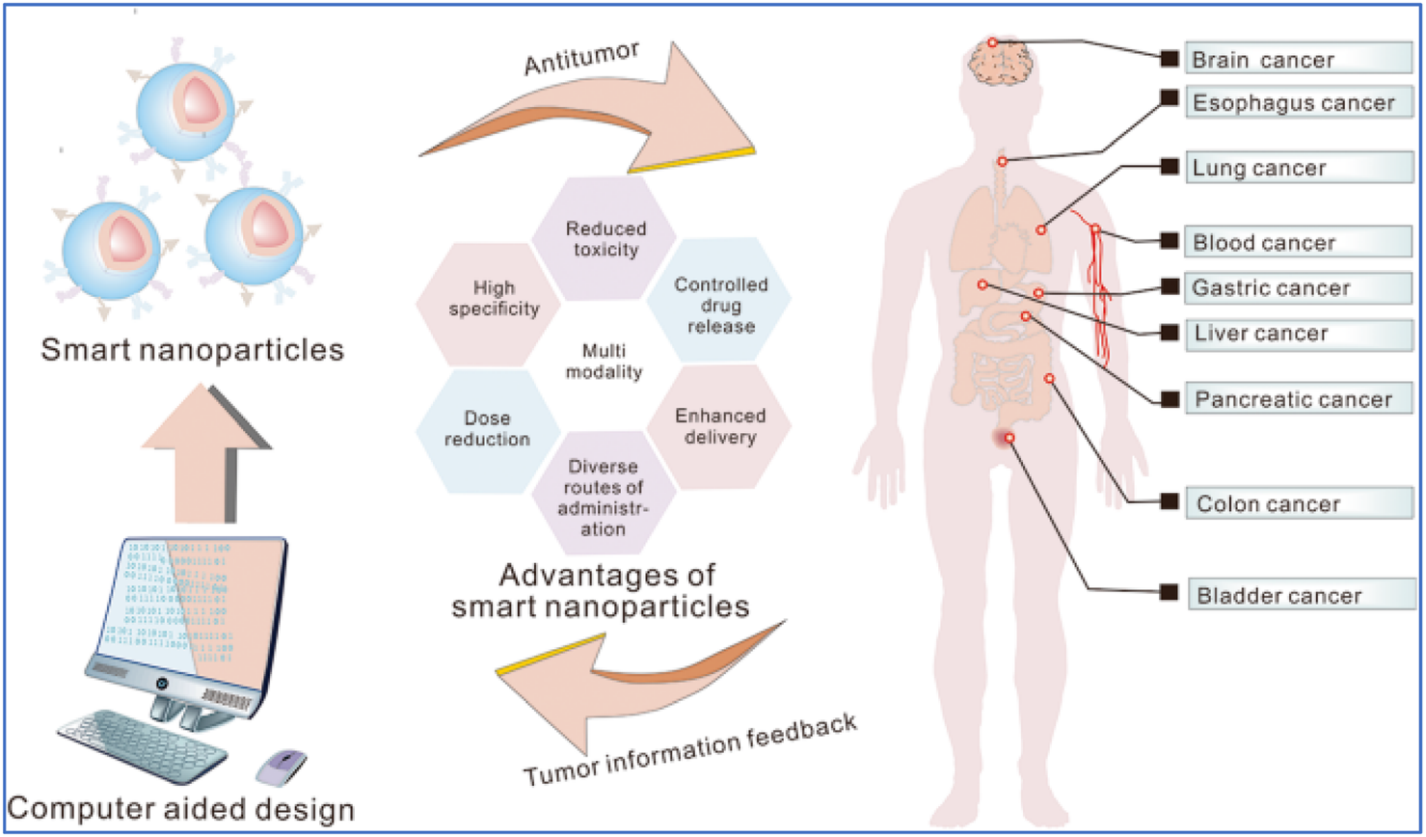
Schematic of the smart nanoparticles functioning in the cancer treatment (adapted with permission from ref. 183. Copyright Springer Nature (2023)).

**Fig. 9:**
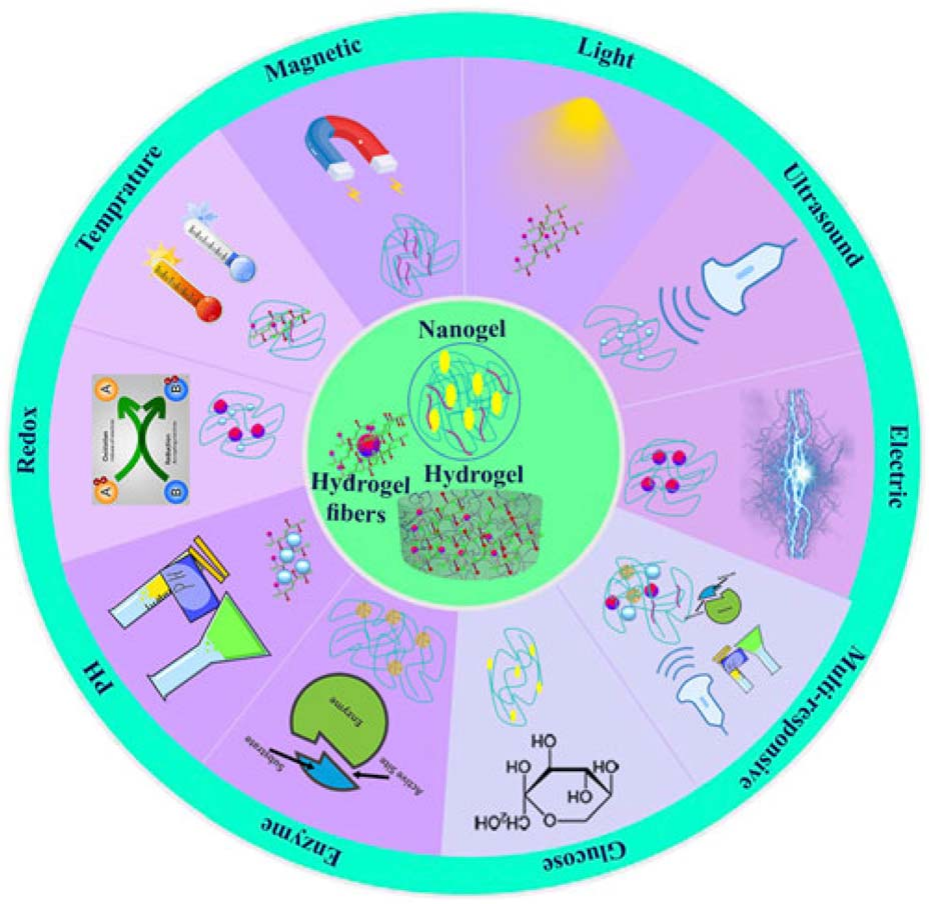
Categories of the stimuli-responsive hydrogels reproduced from ref. 182, Frontiers publisher, (2024).

For the investigation and therapy of breast tissue or intracellular compartments, stimuli-responsive hydrogels offer a comprehensive toolkit. Hydrogels that respond to natural stimuli possess several characteristics, including morphological, structural, mechanical, swelling, drug release, and responsive elements. The study briefly employed electron microscopy methods to ascertain morphological features, such as porosity and roughness. For evaluating structural composition, nuclear magnetic resonance (NMR), Fourier transform infrared spectroscopy (FTIR), and X-ray photoelectron spectroscopy (XPS) were used. Changes in weight or volume were used to assess the swelling behaviors. The viscoelastic properties are determined using a rheometer, and the mechanical and degrading properties of hydrogels are shown by tensile and other test^182^Fig. 8 and 9 represent the stimuli-responsive hydrogels.

In recent years, a novel form of local cancer therapy using hydrogels has been proposed, owing to the favorable results achieved by these formulations in several other biomedical applications. The present review is focused on stimuli-responsive hydrogels (pH-, photo-, ionic strength-, and magnetic-sensitive) proposed in the last 10 years as controlled-release formulations for cancer treatment. Some hydrogels can be sensitive to environmental stimuli such as temperature, pH, light, ionic force, or pressure, suffering sol-to-gel transitions that can allow controlled gelation and drug release at specific sites. Furthermore, non-sensitive hydrogels swell due to water absorption without responding to environmental changes. Mainly, thermo-responsive hydrogels have been widely proposed due to the possibility of having a formulation in the liquid state at room temperature, facilitating its manipulation and injection, which can suffer a transition to a gel state when exposed to body temperature.

#### 3.3.1 Thermoresponsive hydrogels

Hydrogels capable of responding to temperature changes are considered thermoresponsive. For instance, the shift from room temperature (25 °C) to body/tumor temperature (37 °C) can cause thermoresponsive polymer solutions to exhibit a sol–gel phase transition. The temperature at which a polymer solution undergoes a transition from one phase to two phases (an aqueous and a polymer-enriched phase) is designated the ‘critical solution temperature’.^184^

**Fig. 10:**
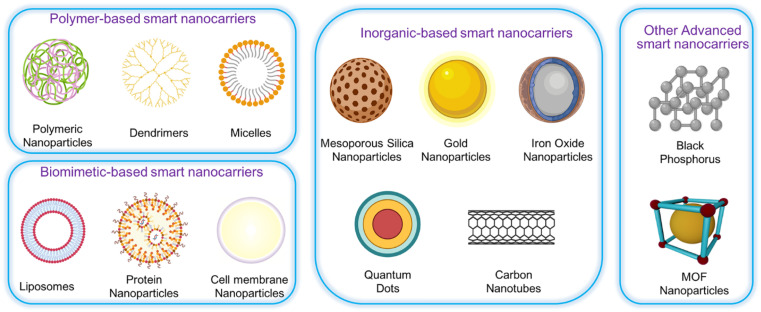
Different kinds of the nanocarriers for smart nanoparticles, (adapted with permission from ref. 183. Copyright Springer Nature (2023)).

The recent advances in typical thermosensitive assemblies and the biomedical applications of thermosensitive hydrogels shows that the alteration in hydrogen bonds induced by temperature, electrostatic interactions, and molecular conformations are the main driving forces for the phase transition of thermosensitive hydrogels. Precision therapies through thermosensitive hydrogels including wound healing, anti-tumors, bone regeneration, buccal, nasal, and ocular diseases have been extensively introduced in diverse medical fields. However, the potential applications are not limited to these categories. For example, some skin diseases such as psoriasis with irregular surfaces, microneedles, and traditional hydrogel sheet patches are not suitable for these uneven or large areas of skin disease.^185^Fig. 10 shows versatile carriers for smart nanoparticles.

#### 3.3.2 Photo-responsive hydrogels

These hydrogels are typically based on polymers containing a photoreactive moiety that responds to light by way of a photochemical reaction, such as cleavage and isomerization. As an example, an injectable self-assembled DOX-loaded hydrogel was prepared based on a four-arm star polymer, PEG-poly(γ-*o*-nitrobenzyl-l-glutamate). The presence of *o*-nitrobenzyl groups in the polymer backbone rendered hydrogels photodegradable by irreversible photocleavage of *o*-nitrobenzyl ester linkages.

#### 3.3.3 pH-Responsive hydrogels

Referring to acidosis of tumor tissues, two strategies have been reported for the design of pH-responsive hydrogels for IT drug administration: (i) the selection of pH-responsive polymers with numerous weak basic (*e.g.*, amine) groups attached to the hydrophobic backbone, thereby being considered polybases (cationic); or (ii) the incorporation of acid-labile linkages within the polymer network or between the polymer and drug, the cleavage of which enables the release of entrapped or anchored drug.

To achieve photo-response, photothermal conversion agents have been widely applied in developing self-healing hydrogels. MoS_2_ nanosheets, as excellent near-infrared (NIR) photothermal conversion agents, can induce high temperatures and contribute to not only the ablation of residual tumors but also a rapid healing process when incorporated into polyvinyl alcohol (PVA) hydrogels under NIR irradiation. An ideal hydrogel implant for post-surgical cancer therapy should be remoldable to match the irregular tissue surface of the resection site.^186^

#### 3.3.4 Redox-responsive hydrogels

The basic principle of designing reduction-responsive hydrogels is the incorporation of disulfide linkages directly into polymers or *via* disulfide-containing crosslinkers, namely, cystamine and its derivatives. Disulfide bonds can be cleaved in the presence of a reducing agent, such as GSH, which, acting as an electron donor, is oxidized to glutathione disulfide. The widely studied Fenton reaction generates free oxygen radicals based on the redox reaction between hydrogen peroxide (H_2_O_2_) and iron. However, the use of H_2_O_2_ and associated environmental issues, along with reaction sensitivity to pH changes, led researchers to search for other possibilities.^187^

#### 3.3.5 Enzyme-responsive hydrogels

Enzyme responsiveness is usually introduced into hydrogels using enzyme-mediated cross-linkages or enzyme-cleavable moieties. The enzymatic degradation of hydrogels drug administration requires the incorporation of an enzyme substrate or substrate mimic, which can be: (i) the polymer itself in the case of biodegradable polymers, such as polysaccharides and polyesters, through the breakage of glycosidic and ester bonds, respectively; or (ii) a peptide linker specifically recognized. The development of a peptoid-peptide drug delivery system to develop a fully soluble formulation capable of undergoing enzyme-responsive hydrogenation *in situ*, triggered by the presence of endogenous phosphatase enzymes within the subcutaneous skin space.^188^

#### 3.3.6 Dual and multiple stimuli-responsive hydrogels

Dual or multiple stimuli-responsive hydrogels respond in a specific and distinct manner to two or more stimuli available in the tumor microenvironment (TME) and/or assisted by an external source. Compared with their single stimulus-responsive counterparts, these hydrogels offer greater control over their responsive behavior and an opportunity to improve the overall performance and applicability to drug delivery. The progress of dual-stimuli-responsive and multi-stimuli-responsive systems that combine multiple response functions in a single system has attracted increasing attention. The inclusion of two or more responsive moieties within the polymer increases the reactivity of nanogels. Incorporating multiple stimulation triggers into a single nanogel delivery system can increase the level of precision in the application of a desired treatment or therapy.^189^

#### 3.3.7 Anisotropic and self-healing hydrogels

There are interactions between metal ions and ligating atom-ended polymer chains with limited branches. Very recently, we have proposed dynamic thiolate-Au (RS-Au) coordination from gold nanoparticles (NPs) as an efficient crosslinking mode for tough and self-healing gels.^190^ Moreover, similar modes have been extended to other precious metals such as silver, for the construction of functional composites.^191,192^ Combined with the easy assembly feature of precious metal nanostructures when exposed to external stimuli, unique metal nano-assemblies would provide numerous possibilities in the creation of soft materials with anisotropic structures.^193^

Anisotropic nanocomposite hydrogels composed of highly ordered lamellar precious-metal nanostructure assemblies (*e.g.* Ag, Au, Pt, and Cu) through the self-assembly of thiolate-modified metal nanostructures as highly branched cross linkers for *in situ* polymerization. As a typical demonstration, in addition to the tough strength, the silver NP/polyacrylamide (PAM) (SNPP) hydrogel delivers an impressively anisotropic mechanical performance with the tangent modulus parallel to the lamellae 3.9 times the perpendicular direction arising from the entanglement of PAM network around the lamellar silver assembly architectures *via* RS-Ag coordination. Additionally, remarkable anisotropy in optics and swelling/deswelling deformations is observed.^194^

### 3.4 Micromotors for drug delivery

Micromotors represent the next generation in nanomedicine since they possess the capability of movement through biological fluids and perform a targeted drug delivery with high accuracy.^195,196^ These microdevices, typically no larger than a human cell, may well change the face of drug delivery as we know it today by offering levels of precision and control that have not been possible earlier. Micromotors can be actuated through various mechanisms such as chemical reactions and physical simulation using external magnetic fields.^197^ Magnetically driven micromotors provide enormous specificity for their motility and targeting in addition to the possible use as hyperthermia agents.^198^ Ultrasound simulation for targeted drug delivery using micromotors is also an effective way. The conversion of ultrasound waves into mechanical energy by ultrasound-driven micromotors therefore presents a non-invasive controllable propulsion method.^199,200^ Photocatalytic micromotors use light to advance certain chemical reactions due to which the movement occurs; this is a controllable method with very high spatial and temporal resolution.^196,201,202^ They can be guided directly to the tissue that is diseased or damaged, thus providing precision delivery of the therapeutic agent at the site of action, with minimal side effects. This improves the therapeutic effectiveness. Technically, micromotors can be designed to release their charge of the required drug in response to particular internal stimuli such as pH levels, temperature, or the presence of certain enzymes, ensuring that drugs are released at the right time and place.^203–206^ These smart devices are also able to deliver chemotherapeutic agents right inside the tumor cells, reducing damage to healthy tissues and thereby possibly decreasing side effects. They can carry antibiotics right to the site of infection, increasing the concentration of the drug at the target site and enhancing treatment outcomes.^203,207–209^

Despite the promising potential, several challenges remain in the development and implementation of micromotors for drug delivery. Biocompatibility is an important issue to be addressed. For the safe use of micromotors inside the human body, the nontoxicity of micromotors and absence of adverse immune response become imperative, along with control and precision of delivering the drug to the desired site. It is tough to envisage the exact control of movement or function of micromotors inside the human body.^210,211^ The achievements in material science, nanotechnology, and biomedical engineering will be of great importance for realizing the given potential of micromotors in drug delivery.

## 4. Manipulating cancer using biosensors

### 4.1 Application of biosensors in cancers

Potentially, one of the most useful instruments for early cancer detection, assessing how well a malignancy responds to chemotherapy and tracking the course of the disease are cancer biomarkers.^212^ Biomarkers can be found in or on tumor cells, although they are usually found in human fluids including blood, serum, urine, or cerebral spinal fluid (Table 1).^213^ Biosensors are devices that look for biological analytes in the human body, such as cancer biomarkers and transform a biological component into a measurable electrical signal.^216^ Biosensors allow quick and precise cancer diagnosis, trustworthy cancer cell imaging, and metastasis tracking by identifying new cancer biomarkers and assessing the efficacy of medications at many target sites.^216^ Biosensors can be used for pathogen detection, cancer diagnosis, and blood glucose monitoring in diabetics. The identification of dangerous bacteria in the air, water, or food is one of the environmental uses of biosensors.^216^ Thus, biosensors can determine whether a tumor is present, whether it is malignant or benign, and whether treatment has been successful in removing cancerous cells by assessing the amounts of specific proteins that are expressed and secreted by tumor cells. Additionally, as most cancer types involve numerous biomarkers, biosensors that can detect many analytes may prove very helpful in the diagnosis and monitoring of cancer. A biosensor's capacity to screen for several indicators expedites diagnosis and improves patient care.^213^

**Table 1:** Biomarkers for cancer detection^213–215^

Type of cancer	Biomarker
Breast	BRCA1, BRCA2, CA 15-3, CA 125, CA 27.29, CEA, NY-BR-1, ING-1, HER2/NEU, ER/PR
Colon	CEA, EGF, p53
Esophageal	SCC
Liver	AFP, CEA
Lung	CEA, CA 19-9, SCC, NSE, NY-ESO-1
Melanoma	Tyrosinase, NY-ESO-1
Ovarian	CA 125, HCG, p53, CEA, CA 549, CASA, CA 19-9, CA 15-3, MCA, MOV-1, TAG72
Prostate	PSA

**Fig. 11:**
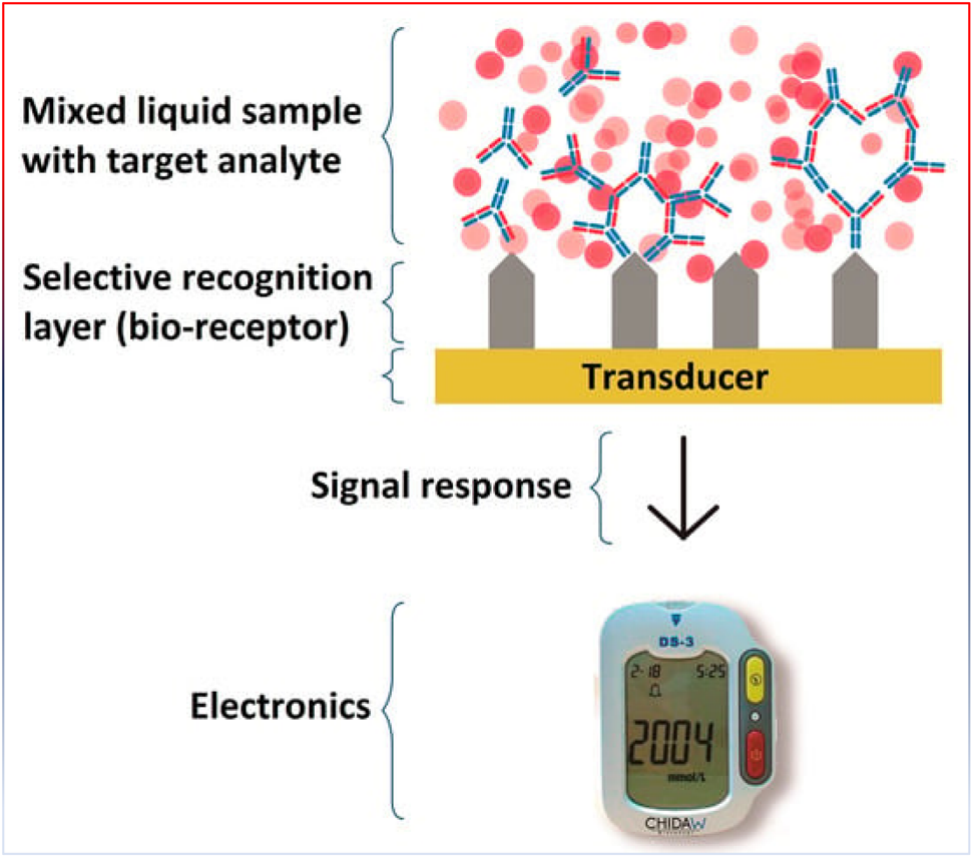
General scheme of a biosensor, reproduced from ref. 217. Copyright, MDPI publisher (2017).

**Fig. 12:**
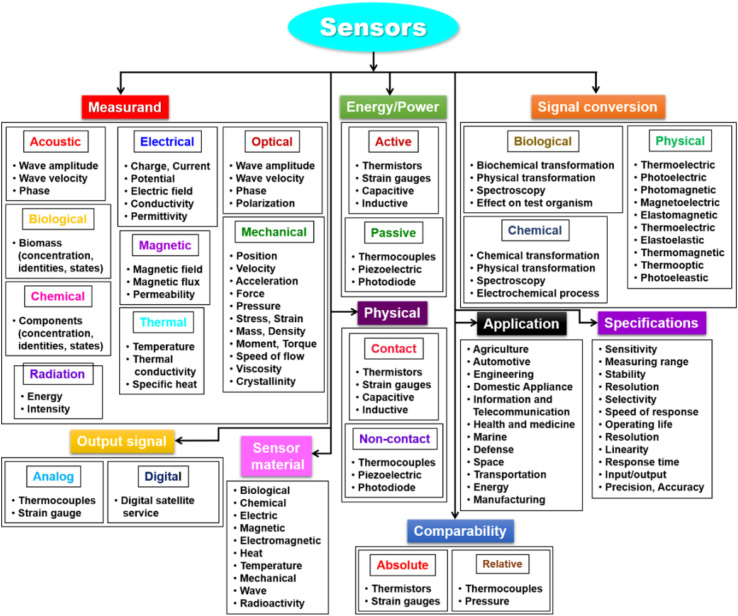
Types of biosensors (adapted with permission from ref. 222. Copyright MDPI publisher (2021)).

Three parts make up a biosensor (Fig. 11): a recognition element, a signal transducer, and a signal processor that displays the results. The molecular recognition component detects a ‘signal’ from the environment in the form of an analyte, and the transducer then converts the biological signal to an electrical output.^218^ The biosensor's recognition element is one of its most important parts. Receptor proteins, antigens, antibodies, enzymes, and nucleic acids are a few examples of recognition elements. Among the fastest detection systems are those that use antigen- and antibody-based recognition elements. An anti-PSA antibody serves as the recognition element for PSA biosensors, which are among the most popular biosensors used in clinical settings to identify prostate cancer. Anti-PSA recognition elements have been connected to surface plasmon resonance-based sensors and microcantilever-based transducers, which employ changes in vibrational frequency following PSA binding to antigen.^219,220^ Transducers are the second component of biosensors. The chemical signal is transformed by the transducer into an electric or digital signal that can be measured, shown, and examined. Transducers fit into four general categories: electrochemical (*i.e.*, amperometric and potentiometric), optical (*i.e.*, colorimetric, fluorescent, luminescent, and interferometric), mass-based (*i.e.*, piezoelectric and acoustic wave), and calorimetric (temperature based).^221^ Nowadays, electrochemical biosensors are the most widely used type of biosensors because of their affordability, simplicity of use, and compact size. Point-of-care (POC) devices can be made from electrochemical biosensors. An electrochemical biosensor is the glucose sensor.^213^ Major types of sensors are represented in Fig. 12.

Potentiometric and amperometric biosensors are among the most popular types of electrochemical biosensors. Ion-selective electrodes are used by potentiometric biosensors to identify an electrical response in the chemical recognition element.^218^

Although it is not yet in clinical usage, a light-addressable potentiometric sensor (LAPS) coupled to a phage recognition element is demonstrating considerable potential in the field of cancer diagnosis.^223^ The phage-LAPS were able to detect the cancer biomarker hPRL-3 and cancer cells (MDA/MB231 breast cancer cell line) with high sensitivity. Additionally, the current generated when a potential is applied between two electrodes is measured using amperometric transducers. A current that is produced by oxidation or reduction reactions can be monitored. Sequence-specific DNA serves as the recognition element of amperometric-based biosensors for cancer detection, which show great promise for cancer diagnosis.^224^ These sensors recognize and hybridize certain DNA sequences found in the genomes of malignant cells, allowing them to identify the presence of gene abnormalities linked to cancer. It was with this kind of technology that Wang and Kawde made a breakthrough,^225^ which used chrono-potentiometric transduction biosensors to determine that mutations of BRCA1 and BRCA2 associated with hereditary breast cancer.

Asphahani and Zhang^226^ demonstrated the feasibility of a cell-based electrochemical biosensor that measures changes in cell impedance in response to analyte. These cell-based biosensors, also known as cytosensors, use biological sensing elements, namely, live cells, to track changes brought about by different stimuli. This type of sensor can be used to track how anticancer drugs affect the molecules that they target in cancer therapy. The majority of anticancer medications kill cancer cells by inducing apoptosis through p53. The cell experiences significant alterations in its shape overall, ion channel permeability, and membrane integrity during apoptosis. A cytosensor, as opposed to a sensor that employs a pure component (such as an enzyme or receptor), would be able to identify these cellular alterations, allowing for a more precise assessment of the anticancer agent's pharmacological efficacy.

Moreover, optical biosensors are light-based sensors that track variations in particular light wavelengths. The transducer may be based on colorimetric, fluorescence, luminescence, or interferometry. Optical transducers provide an electrical or digital readout by converting wavelength changes or SPR in response to analyte recognition.^213^ The esophageal laser fluorescence-based optical biosensor for the diagnosis and monitoring of throat tumors is another fascinating example of this type of technology in cancer detection. The device targets the surface of the esophagus with a laser beam that emits a particular wavelength of light once the patient has ingested it. Certain wavelengths of light are reflected by the esophagus wall depending on whether the tissue is made up of normal or malignant cells. After more than 200 patients underwent testing, it was shown that this sensor could detect cancer more than 98% of the time.^227^ By using this type of biosensor, surgical biopsies and the pain and recovery time associated with them could be avoided. Zhang and colleagues^228^ created a biosensor based on nanowires to identify micro-RNAs (miRNAs). miRNAs are crucial modulators of gene expression and have been linked to the emergence of cancer. Conventional techniques for identifying miRNAs such as northern blot analysis are expensive, time-consuming, and have poor sensitivity. The use of miRNAs as cancer biomarkers has advanced with the creation of a sensitive, affordable, and user-friendly biosensor for identifying miRNAs linked to cancer.

### 4.2 Carbon-based biosensors

Certain biosensors are constructed using nanomaterials, such as carbon nanotubes (CNTs), which are among the most widely used NMs in biosensing, tissue engineering, and drug delivery. They are hollow cylindrical tubes that are made up of one, two, or more concentric graphite.^229^ For the extremely sensitive detection of analytes in healthcare, industry, environmental monitoring, and food quality screening, CNT-based biosensors and diagnostics have been used. Electrochemical sensing has been their application, primarily for glucose monitoring but also for the detection of other biomolecules such as cells, microorganisms, DNA, and other biomarkers.^229^ Due to its fascinating qualities, graphene, an atomically thin layer of sp^2^-hybridized carbon, is also one of the most widely employed NMs for biosensors. Quantum Dots (QDs) are also inorganic nanocrystals that possess special optical characteristics such as limited size-tunable emission spectra, broad excitation, and strong photochemical stability. They have been widely employed in the development of optical biosensors to detect ions, chemical compounds, pharmaceutical analytes, and biomolecules such as proteins, amino acids, carbohydrates, enzymes, and neurotransmitters. They have also been used to identify cancer target areas *in vivo*.^229^ For this reason, the development of biosensors has truly benefited greatly from the application of nanotechnology, such as quantum dots and nanoparticles.

Therefore, one of the major scientific, engineering, and educational problems of the twenty-first century is the development of ultra-sensitive biological and chemical sensors. To address the demands of various industries such as pharmaceutical discovery, pathogen detection, and *in vitro* medical diagnostics, the next-generation biosensor platforms need to achieve notable advancements in sensitivity and specificity. The most advanced diagnostic biosensors available today are built on various technologies, most commonly the polymerase chain reaction (PCR) amplification of a sample with the right primers and detection techniques, or the enzyme-linked immunosorbent assay (ELISA). Biosensors of the future could potentially be chip-sized devices worn on the body to track vital signs, diagnose anomalies, or even indicate an emergency need for assistance.

### 4.3 MOF-derived biosensors for detecting cancer biomarkers

**Fig. 13:**
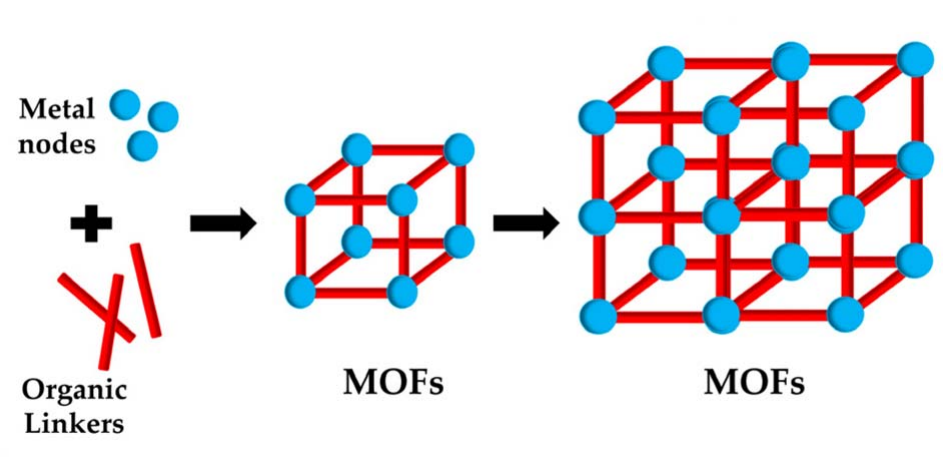
Schematic of MOF fabrication (adapted with permission from ref. 230. Copyright, MDPI publisher (2023)).

Metal–organic frameworks (MOFs) are a new class of porous coordinated polymers considered to be inorganic–organic hybrids composed of multidentate organic ligands and inorganic metal nodes linked together *via* coordinate covalent bonds known as struts (Fig. 13). They have distinguished remarkable research interest owing to their unique periodic network structures of crystalline porous materials with well-defined tunable pore size, high thermal stability, superior catalytic activity as well as large specific surface area and customizable structure. The flexibility of the organic materials along with the rigidity of the inorganic materials makes MOFs demonstrate enormous prospects in modern material science exhibiting different applications in various fields such as gas sorption/separation, catalysis, drug delivery, sensing, and biomedical imaging. Additionally, the wide varieties of selecting the organic ligand and the metal centers provide great flexibility for MOF synthesis with various physical and chemical characteristics and desirable structure, pore size, and morphology.^230–235^

Different forms of MOFs can also be utilized as a matrix to enhance the electrical, chemical, or optical signs, to attach the biorecognition materials covalently or physically. In this regard, MOF-based biosensors have many advantages for biosensing compared with other sensors: (a) MOF-based biosensors exhibit low toxicity, high thermal stability in different environments, simple one-pot fabrication mechanism, reasonable synthesis cost, and good biodegradability.^112^ (b) MOFs have a wide variety of functional groups that can be functionalized easily. (c) In addition, MOFs can form hydrogen bonds and π–π stacking with the capture probe molecule. (d) They have striking quenching characters that enhance the detection sensitivity. The defined porous and crystalline structure of the MOF conformation, which makes them exceptional key molecules for fabricating nanostructures *via* high-temperature pyrolysis.^236–238^ Compared to other methods applied for nanostructure fabrication, MOF pyrolysis can integrate different functionalities in one step and control the composition, size, shape, and structure of the nanostructures. In general, three types of nanostructure-based MOFs can be fabricated: MOF-derived inorganic metal oxides, composites with MOF-based metal oxides, and carbon material-incorporated metals.^239^ Nanostructure-based MOFs exhibit significant mechanical and chemical stability, tunable pore size, porous nanostructure, and large surface area, which make them promising candidates for use as highly potent biosensors. Owing to their favorable characters for detecting various very small analytes in complex solutions, MOFs and their based composites have gained great interest in developing and performing electrochemical and optical biosensors.^237,238,240^

The early detection of cancer has a great impact in controlling the disease and reducing cancer mortality significantly to save a life; consequently, great efforts have been exerted to develop new methods and techniques for the early detection of the signs of this disease. Cancer biomarkers are specific macromolecules that are usually spread in the cancer tissues or the body fluids as an indication of the presence and development of cancer. Cancer biomarkers can be genetic materials, whole cells, or biomolecules. For detecting different types of cancers, there are more than 160 biomarkers. One of the most common and important cancer biomarkers is carcinoembryonic antigen (CEA), also known as CD66e or CEACAM5. Its overexpression is related to ovarian, colon, breast, and lung cancer.

Recently, MOFs have been used intensely to enhance the selectivity and sensitivity of biosensors for the early detection of cancer biomarkers. The unique features of the MOFs like huge surface area, hierarchical structure, uniform porous crystalline structure, and geometric configuration facilitate the interaction with various analytes and allow for more potential immobilization of bioreceptors such as antibodies and enzymes onto the surface of the DNA probes. The large surface available for immobilization allows more bioreceptors to be attached to the target analyte, leading to higher sensitivity for the detection of the target analyte (cancer biomarker).

In the past few years, different research papers have described the development of novel MOFs-based biosensors for the early detection of biomarkers through various techniques such as optical, electrochemical, and photoelectrochemical processes. Xingxing Zhou *et al.*^241^ have developed electrochemical impedimetric aptasensors for efficient detection of the carcinoembryonic antigen proteins using Pt@CuMOFs-hGq-GOx as signal transduction probes. Pt@CuMOFs were applied as potential nanocarriers for immobilizing the Pt nanoparticles, the glucose oxidase (GOx), and the hemin/G-quadruplex (hGq). The results revealed that the electrochemical impedimetric signal (EIS) was significantly amplified and the electron transfer was hindered in the sensing interface, which enhanced the sensitivity of the carcinoembryonic antigen (CEA) aptasensor with 0.023 pg mL^−1^.^241^

Liu and his research group^242^ have successfully developed a novel electrochemical immunoassay for the detection of CEA using silver(i)-terephthalate MOFs containing gold nanoparticles as a signal unit. The results they obtained demonstrated that the developed Ag MOF-decorated electrode was able to detect the target analyte CEA with a low limit of detection (LOD) of 8.0 fg mL^−1^, and Ag(i) was detected from MOF signals directly without preconcentration and acid dissolution, which reduced the detection time and simplified the detection steps. Moreover, the modified voltammetric immunoassay revealed a wide linear range along with a low detection limit, indicating enhanced analytical performance.

**Fig. 14:**
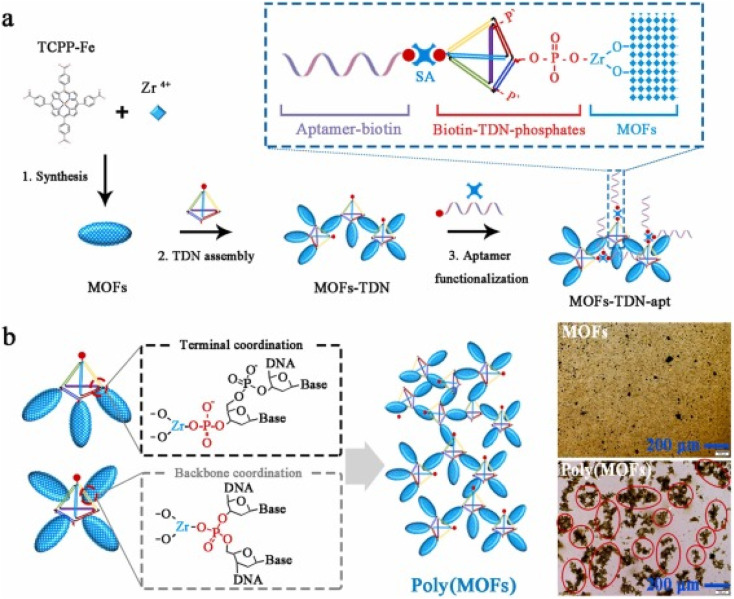
(a) Synthesis of MOFs and aptamer functionalization. (b) Principle of controlled assembly of MOFs induced by tetrahedral DNA nanostructure (TDN)^243^ (reproduced with permission from ref. 243. Copyright Elsevier (2024)).

Otherwise, Q. Liang *et al.*^243^ and coworkers reported a novel method for developing a simple colorimetric biosensor for efficient detection of the human epidermal growth factor receptor 2 (HER2)-positive breast cancer. The MOF assembly process and the functionalization of the interface are shown in Fig. 14. First utilizing TCPP-Fe and zirconium ions, PCN-222 (Fe) was prepared as a type of MOF-derived nanomaterial.^244^ Then, tetrahedral DNA nanostructure (TDN) decorated with phosphate groups was loaded onto the surface of the as-prepared MOFs to induce the MOF self-assembly to afford the desired polymeric MOF-TDN superstructure complex. Finally, streptavidin was applied to connect the biotin-modified DNA aptamer to the TDN containing the biotin-modified vertex, affording the aptamer modification of the poly(MOFs) (MOFs-TDN-apt).^245,246^

In general, novel developed colorimetric biosensor using DNA framework-controlled poly(MOFs) revealed significant analytical performance with high selectivity in diagnosis of the HER2-positive breast cancer. The protein detection was achieved at the molecular level with a limit of detection (LOD) of 12 pg L^−1^ and cellular level with an LOD of 10 cells.^243^

### 4.4 Biomarkers

Biomarkers are commonly named molecular markers or/and signature molecules. There are different definitions for the term “Biomarkers”. The term biomarker/s can be identified as “an indicator of normal biological processes, pathogenic processes, or a pharmacological response to a therapeutic intervention”. Also, biomarkers can be defined as “A biological molecule found in blood, other body fluids, or tissues that is a sign of a normal or abnormal process, or a condition or disease”. Hence, using biomarkers in the medical field have evolved a great interest in different applications. It can help in measuring the body's interaction with a specific disease or medication. It motivates the recognition of the health level of different organisms. Using biomarkers is a promising technique for cancer detection, evaluation, and correlated cancer treatment responses.^247–250^ To identify the proper cancer biomarkers, different categories were involved. One of the interesting biomarker classification was reported by Mishra and Verma, 2010.^251^ Their classification reveals that cancer biomarkers can be divided into three main categories based on the status of the disease, biomolecule biomarkers (such as DNA, RNA, and protein), and other criteria (where the pathological biomarkers and imaging biomarkers were reported).

The biomarkers can be classified into different categories based on disease states. These categories include the prediction, detection, diagnostic, and prognostic biomarkers. The prediction biomarkers may be used to predict the developing status of the cancer.^252–254^ They can help identify individuals who are at a higher risk of developing cancer and may benefit from early screening or preventive measures, while the detection biomarkers are useful in the detection of cancer presence. Hence, the detection biomarkers can be used for early diagnosis and screening purposes. Then the diagnostic biomarkers are commonly used for the confirmation and determination of the present cancer type and stage.^255,256^ Those biomarkers can provide a great insightful guide on further treatment decisions and prognosis. Lastly, the prognostic biomarkers target the assessment of the prognosis. They can reveal information about the disease status progress, and the response to specific treatments.

Notably, some biomarkers are overlapping in nature, where they can be effective for different categories.

The biomolecule biomarkers can be categorized into DNA, RNA, protein, and glyco biomarkers. Single nucleotide polymorphisms (SNPs) are considered the main DNA biomarker.^257^ While the RNA and MicroRNA (miRNA) biomarkers in a tissue- and time-dependent manner could be an indication of the cancer's type.^258,259^ Furthermore, the protein biomarker is an important biomolecule biomarker due to its relevance to the status of the disease due to its ability to affect the routes in the cells.^251,260,261^ Different proteomics were used in different cancer types of detection including prostate cancer^262^ and leukemia;^263^ the biomolecule biomarkers can provide insights into different types of gene mutations, protein expression, and an indication of disease presence.

Based on Mishra and Verma (2010),^251^ the third category in their reported biomarker classification included other criteria such as pathological biomarkers and imaging biomarkers. The pathological biomarkers can be divided into two main categories: the viral and bacterial markers. It was reported that viruses can be an attractive biomarker depending on the present cancer type.^260,264^*i.e.* Hepatitis E may considered a consistent biomarker in the presence of acute hepatitis E infection,^265^ while viral hepatitis B and C may be potential effective biomarkers of hepatocellular carcinoma/liver cancer. Similarly, the bacterial biomarkers reveal a correlation between the bacteria type and the disease where *Helicobacter pylori* was considered as a determined biomarker for gastric cancer.^266^ Biomarker research is gaining great interest in the medical field through the detection of new markers and their relevant potential applications (Table 1).

### 4.5 Label-free biosensors

The term “biosensor” refers to a detecting device where a receptor molecule binds to the specific analyte and a transducer.^267,268^ The role of the transducer is to convert the received signals into computable quantities. It is difficult to detect the organic analytes *via* the transducer due to their complex nature. Hence, different labels can be used to facilitate this step. This includes the use of fluorescent markers or radioactive molecules. As a result, there will be a direct proportion between the final sensor signal and the label's number. Based on that, the biosensors can be categorized into labeling and label-free sensing. The disadvantage of using a label sensor is that it is time-consuming compared to a label-free sensing, and there is the possibility of a negative interaction effect between the receptor and the analyte. Various label-free sensor categories depend on the working principle, the used substrate, and lastly the recognition probe.^267^

The optical-based label-free biosensor is considered as an example of the working principle sensor, where it depends on the analyte's optical transduction. The mechanism of work for such biosensors relies on the connection between biological molecules and the transducer, where the disturbance in the analyte will be measured as optical change measurements. The optical-free biosensor is applicable for the qualitative detection of any biological additives in the tested samples.^269,270^ It can be used for the detection of DNA-oligomer hybridization due to surface reflection. The sensor can also be used in the quantification of DNA-oligomer hybridization through the measurements of the reflected light intensity, facilitating tracking of any possible sequence changes as an indication of cancer disease or as a response to specific treatment.^271^

The paper-based label-free biosensor is a represented example of substrate-based sensors, which depends on the paper's capillary filtration and hydrophilic nature,^272^ while the used transducer in the paper-based label-free sensors depends on the electrochemical and piezoelectric properties. This type of sensor is important as a diagnostic tool for emergency environmental evaluation. The presence of different pathogens in water can cause a wide range of diseases. Adkins *et al.* (2017) developed^273^ a paper-based label-free biosensor that depends on the electrochemical. Adkins used the developed sensor in the detection of a bacteria strain, *E coli*, in contaminated water.

The aptamer-based label-free biosensors are the main model of the recognition element-based sensors. The aptamers definition based on Samuel and Rao 2022,^267^ are oligonucleotides of single-stranded DNA or RNA that can be chosen to bind a specific analyte.

## 5. Conclusion and future perspectives

In this review article, the authors deliberately introduced topics related to cancer treatment and therapy. First, a general introduction to the cancer history, determined specifications of the cancer cells and evolution of the facts related to cancer was provided. Then, the familiar symptoms and side effects of the cancer treatment approaches were discussed. The review article was separated into three categories: (1) the start of the review reveals the implication of treatments based on nanoparticles such as magnetite and gold nanoparticles and relatively reported research towards these points. Next to this section, the article surveyed the drug delivery approach applied to the treatment of cancers and its implications, which comprises the core–shell structures and the stimuli-responsive hydrogels such as temperature and pH-triggered hydrogels and core/shell compositions, which specify the delivery of loaded nanoparticles under desired conditions. Finally, the third section of the review article highlights cancer manipulation using various biosensors. This encompasses carbon-based biosensors, metal–organic frameworks-based biosensors and label-free biosensors. The evolution of cancer research is expanding due to the impact of society, human life, and regular activities. The forthcoming progress of cancer research grasps immense potential as speedy developments in technology, and biomedical areas continue to redesign our knowledge and track this multifaceted disease. Cancer research is evolving with methods that provide optimism for more operative treatments, former detection, and ultimately, the possibility of cures. The integration of artificial intelligence into the formulation and implantation of point-of-care devices is essential for the determination of highly efficient drugs that give the most promising results with the lowest concentration, and the drug resistance evaluation is crucial. Moreover, the consideration of the psychological effects on patients in terms of the mental state, fatigue, and other physical symptoms should be delved more. As our thoughts on cancer genomics develop, treatments will upsurge increasingly targeted, lowering the possible side impacts while enhancing the effectiveness. One of the greatest promising fields for prospective cancer research is precision medicine. Personalized therapy, customized to the genetic makeup of a patient's cancer, has displayed noteworthy promise. Future research will most likely focus on the development of tailored vaccinations, gene editing technologies such as CRISPR, and more complex biomarker-driven medicines that target the distinct characteristics of each malignancy. Immunotherapy, which utilizes the body's immune system to combat cancer, is another direction primed for enormous discoveries. Future immunotherapy will inspect new methods to improve the immune system's capability to diagnose and terminate cancer cells. The conjugation of immunotherapy with subside treatment options such as chemotherapy, radiation, or targeted therapies may cause a more plausible and long-lasting response, particularly for malignancies that are generally problematic to cure. Early detection and diagnosis facilitate potential contribution in cancer research. Liquid biopsies, which analyze the biomarkers in the blood samples to sense the cancer cells at initial stages, are developed and formulated. The upcoming research might afford a non-invasive test which might screen several cancers during a single blood draw, allowing premature intervention when the treatments are most efficient. The incorporation of artificial intelligence and machine learning into the diagnostic apparatus is also probable to advance the precision and the feasibility towards cancer detection. Furthermore, the development in the nanotechnology and drug delivery system devices will push the boundaries towards the administration of cancer therapy. Nanoparticles and other innovative delivery approaches could allow more accurate cancer cell targeting, preventing healthy tissues from damage and elevating the patient outcomes. The learning of the tumor microenvironment will also provide novel insights into the therapeutic approaches to prevent cancer from spreading, and how to evade metastasis, which remains one of the principal reasons for cancer-related fatalities. The performed research on cancer inhibition comprising the vaccines provided for viruses associated with cancer could deliver a pivotal contribution in reducing the incidence of cancer internationally, as well as the enhancement of health treatment protocols. Despite these potentials and advances, the main obstacles that need to be tackled remain as cancer's inherent heterogeneity nature, the upraised drug resistance, and the demand for more equitable access to progressive treatment. Forthcoming research on cancer should attribute not only the technological and therapeutic influences but also the social and economic obstacles that oppose many clients from obtaining the required care. With the evolution of scientific research through multidisciplinary collaborative efforts, the route towards efficient treatment and remedy, earlier detection, and supreme prevention is becoming a clear demand and easier to control. The research in cancer treatment is in progress through continuous efforts by scientists, physicians and other collaborative contributions to fight this disease. We hope that in the near future, this disease will become a usual issue as cold, or usual diseases bringing the world a peaceful and healthier society and pushing technology and mankind's life conditions.

## Data availability

The data presented in this study are available from the corresponding author upon request.

## Author contributions

Z. S. Shaban and E. M. Hieba contributed to the conceptualization and writing of the first draft, explored the logical flow of the review, prepared the drawings, and revised the manuscript. H. A. Batakoushy approved the presented idea and contributed to the writing of the manuscript. H. R. M. Rashdan took part in the writing of the manuscript, approved the manuscript propagation, revised, and edited the manuscript, E. Ismail contributed to the writing of the manuscript, revised the manuscript, S. M. Elkatlawy contributed to the investigation of the manuscript sections, logical flow, and the writing, revision, and editing of the manuscript, A. Elzwawy validated the proposed idea, contributed to the writing – review & editing, the following and updating of the versions, besides guided and supervised the manuscript.

## Conflicts of interest

The authors declare no conflict of interest in this work.
