# Correction: Cancer treatment approaches within the frame of hyperthermia, drug delivery systems, and biosensors: concepts and future potentials

**DOI:** 10.1039/d4ra90156h

**Published:** 2025-01-15

**Authors:** Zeinab S. Sayed, Eman M. Hieba, Hany A. Batakoushy, Huda R. M. Rashdan, Enas Ismail, Saeid M. Elkatlawy, Amir Elzwawy

**Affiliations:** a Faculty of Applied Medical Science, Misr University for Science and Technology (MUST) Giza Egypt; b Chemistry and Entomology Department, Faculty of Science, Cairo University Giza 12613 Egypt; c Department of Pharmaceutical Analytical Chemistry, Faculty of Pharmacy, Menoufia University Shebin Elkom 32511 Egypt; d Chemistry of Natural and Microbial Products Department, Pharmaceutical and Drug Industries Research Institute, National Research Centre 33 El Buhouth St., Dokki Giza 12622 Egypt Hudadawoud20@yahoo.com; e Department of Craniofacial Biology, Pathology & Radiology, Faculty of Dentistry, University of the Western Cape, Tygerberg Hospital Cape Town 7505 South Africa; f Physics Department, Faculty of Science (Girl’s Branch), Al Azhar University Nasr City Cairo Egypt; g Department of Physics, Faculty of Science, University of Sadat City Fifth Zone Sadat Egypt; h Ceramics Department, Advanced Materials Technology and Mineral Resources Research Institute, National Research Centre (NRC) 33 El Bohouth St., Dokki Giza 12622 Egypt elzwawy1@gmail.com aa.elzwawy@nrc.sci.eg

## Abstract

Correction for ‘Cancer treatment approaches within the frame of hyperthermia, drug delivery systems, and biosensors: concepts and future potentials’ by Zeinab S. Sayed *et al.*, *RSC Adv.*, 2024, **14**, 39297–39324, https://doi.org/10.1039/D4RA06992G.

The authors regret that the one of the affiliations (affiliation *e*) was incorrectly shown in the original manuscript. The corrected list of affiliations is as shown above.

The Royal Society of Chemistry apologises for these errors and any consequent inconvenience to authors and readers.

